# Supplementary figures and images for: Estimating the risk of species interaction loss in mutualistic communities
Source: PLoS Biol. 2020 Aug 31;18(8):e3000843. doi: 10.1371/journal.pbio.3000843 (PMC7485972; doi:10.1371/journal.pbio.3000843)

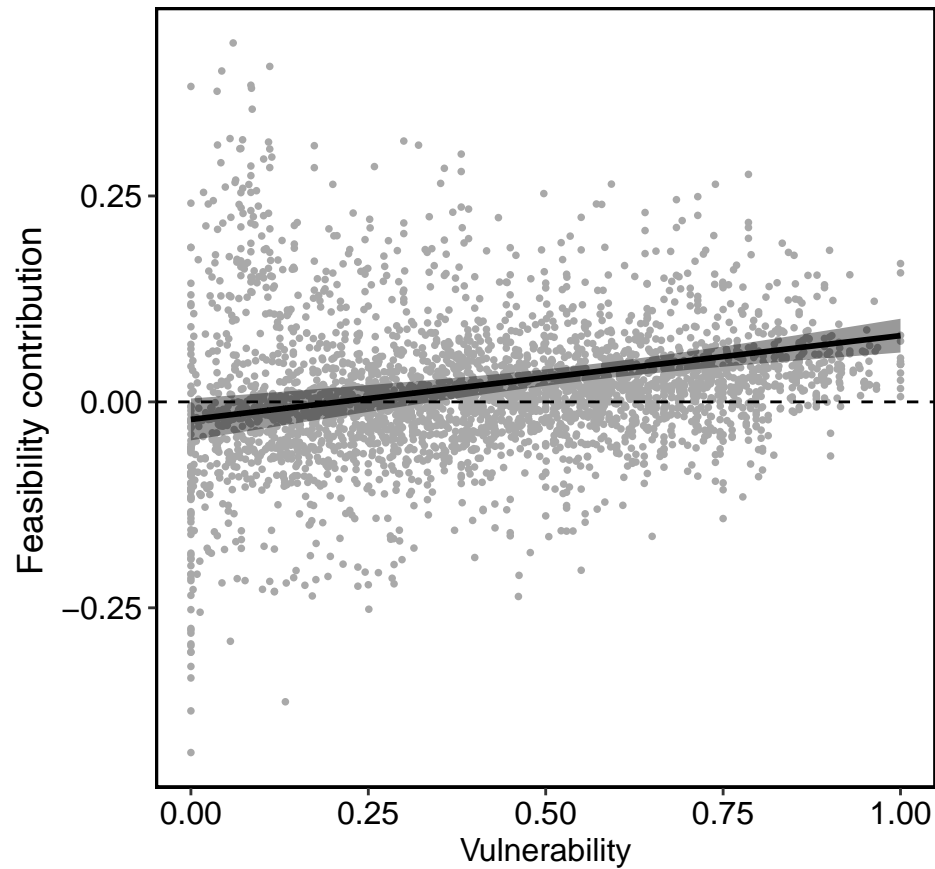

Supplement: S1 Fig — The relationship between vulnerability (the likelihood of a link being lost) and feasibility contribution (the contribution of a link to a network’s feasibility) for all species–species links across 41 mutualistic networks (ρ = 0.01). Best fit line is from a mixed-effects model with feasibility contribution as the response variable, vulnerability as a fixed effect, and network identity as a random effect. Grey band represents the 95% confidence interval. Data underlying this figure are given in S2 Data (https://doi.org/10.6084/m9.figshare.12689258.v1). (PDF) [file pbio.3000843.s001.pdf]

**a**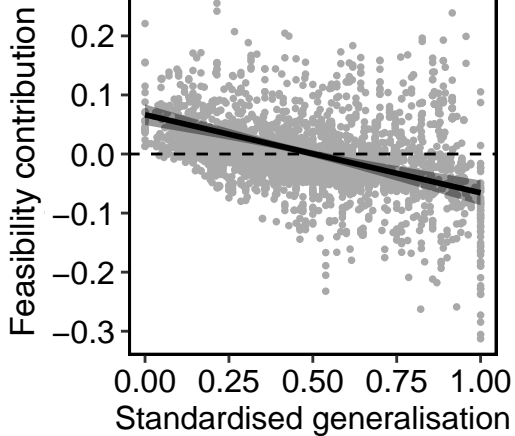**b**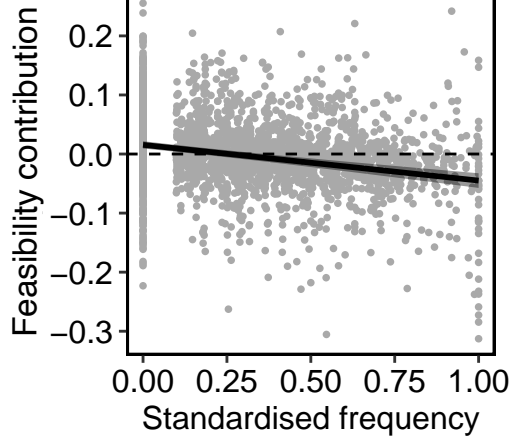**c**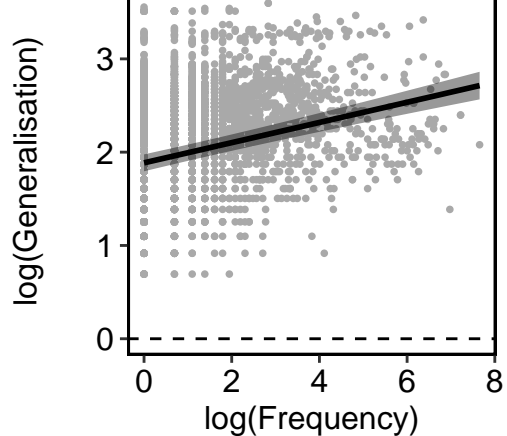

Supplement: S2 Fig — The relationships, for all species–species links across 41 mutualistic networks (ρ = 0), between (a) standardised generalisation and feasibility contribution (Wald test: χ2 = 50.09, df = 1, P ≤ 0.001), (b) standardised frequency and feasibility contribution (Wald test: χ2 = 83.19, df = 1, P ≤ 0.001), and (c) log(frequency) and log(generalisation) (Wald test: χ2 = 125.91, df = 1, P ≤ 0.001). Best fit lines are from mixed-effects models with network identity as a random effect. Grey band represents the 95% confidence interval. Data underlying this figure are given in S3 Data (https://doi.org/10.6084/m9.figshare.12689258.v1). (PDF) [file pbio.3000843.s002.pdf]

# Frequency

(a) Genus

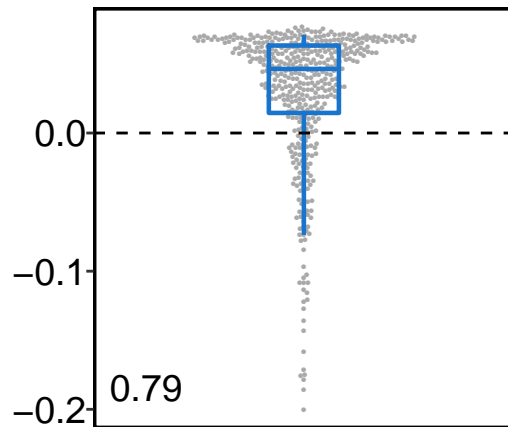

(b) Family

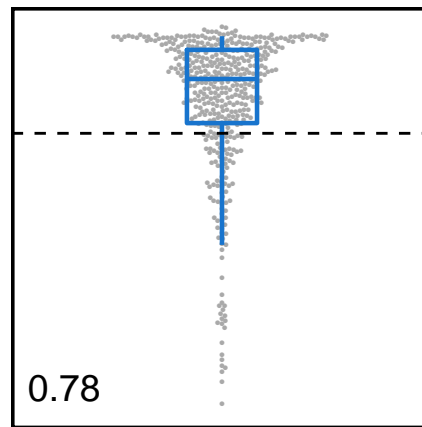

(c) Order

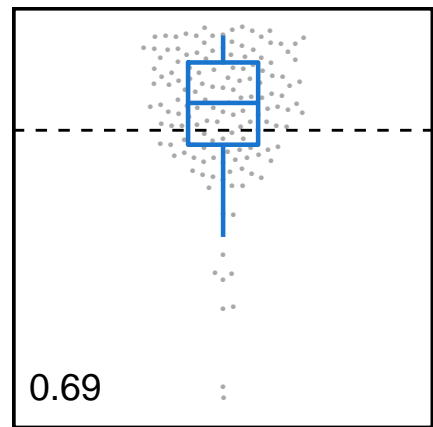

# Generalisation

(d) Genus

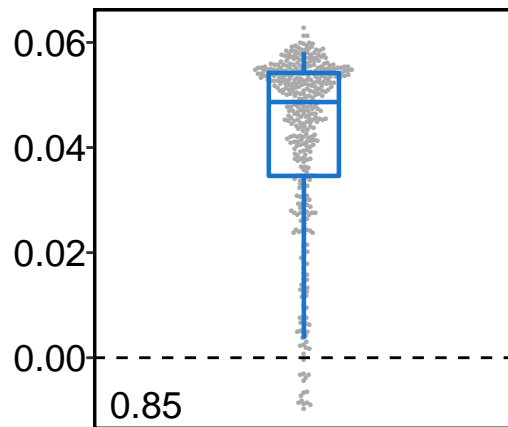

(e) Family

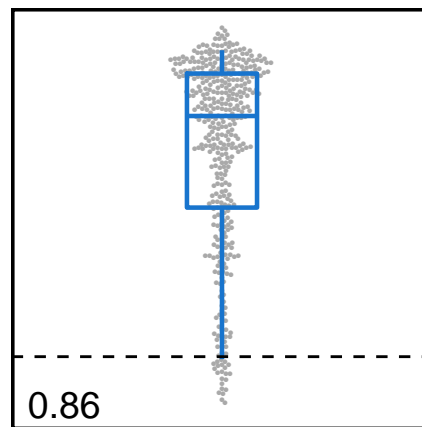

(f) Order

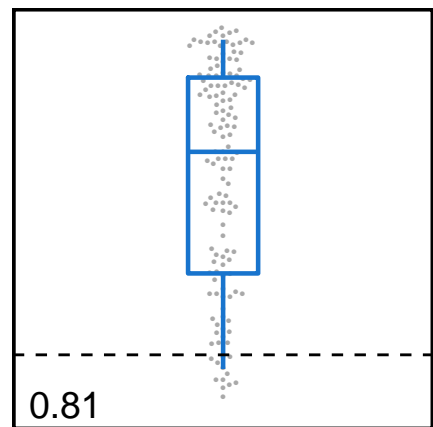

Supplement: S4 Fig — The degree of taxonomic consistency for each interaction at each taxonomic level, for both standardised frequency and generalisation (ρ = 0). Taxonomic consistency is the tendency for properties of an interaction to be more similar across occurrences than expected by chance. Points represent individual interactions. Boxplots represent 5%, 25%, 50%, 75%, and 95% quantiles of the same data, moving from the bottom whisker to the top whisker. Number in bottom left of each panel is the proportion of interactions that exhibited positive consistency (VarianceObserved < VarianceNull). Considering frequency, there was significant taxonomic consistency for 14% of genus, 19% of family, and 21% of order interactions (see Methods). Considering generalisation, there was significant taxonomic consistency for 21% of genus, 25% of family, and 43% of order interactions. For visualisation, a small number of points with low values were removed. The percentage of points with values lower than the y-axis minimum are as follows for each panel: (a) 1.5%, (b) 0.6%, (c) 0.7%, (d) 11.5%, (e) 8.8%, and (f) 10.5%. Data underlying this figure are given in S9 Data (https://doi.org/10.6084/m9.figshare.12689258.v1). (PDF) [file pbio.3000843.s004.pdf]

# Frequency

(a) Genus

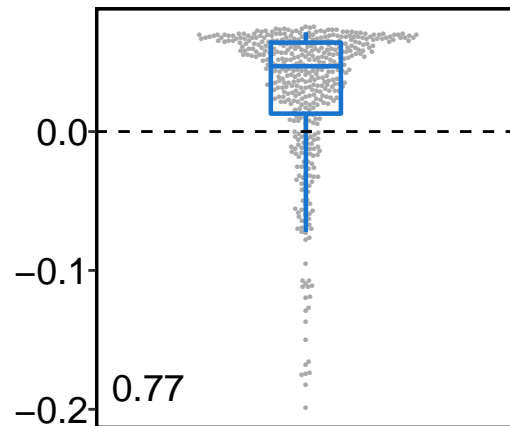

(b) Family

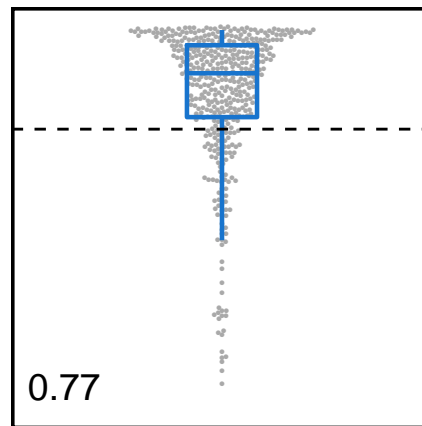

(c) Order

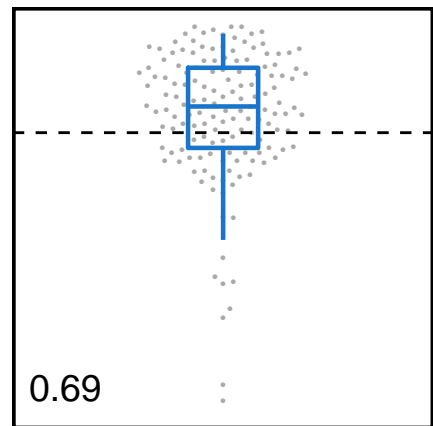

# Generalisation

(d) Genus

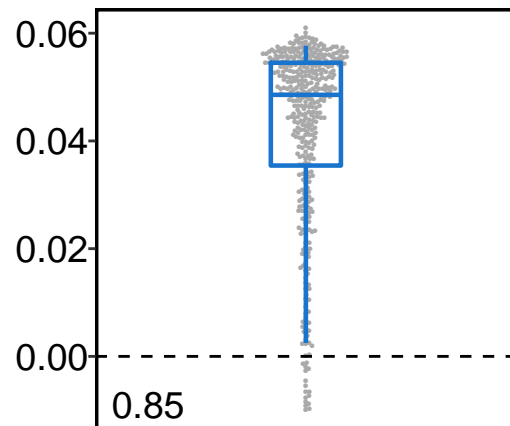

(e) Family

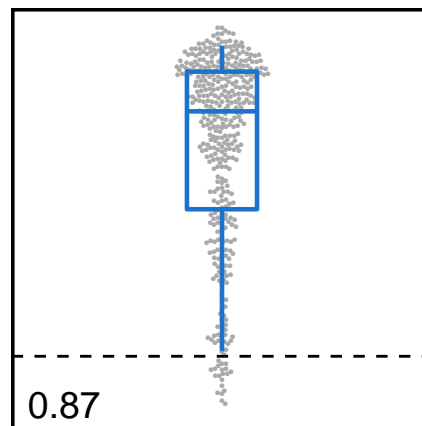

(f) Order

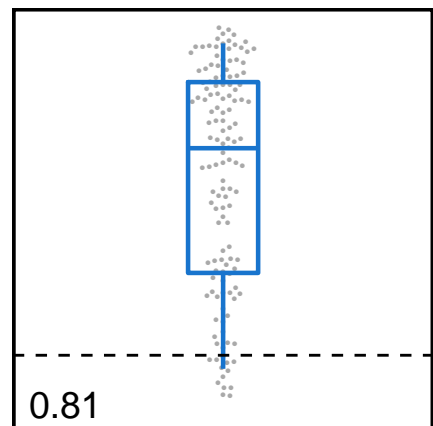

Supplement: S5 Fig — The degree of taxonomic consistency for each interaction at each taxonomic level, for both standardised frequency and generalisation (ρ = 0.01). Taxonomic consistency is the tendency for properties of an interaction to be more similar across occurrences than expected by chance. Points represent individual interactions. Boxplots represent 5%, 25%, 50%, 75%, and 95% quantiles of the same data, moving from the bottom whisker to the top whisker. Number in bottom left of each panel is the proportion of interactions that exhibited positive consistency (VarianceObserved < VarianceNull). Considering frequency, there was significant taxonomic consistency for 14% of genus, 19% of family, and 20% of order interactions (see Methods). Considering generalisation, there was significant taxonomic consistency for 21% of genus, 24% of family, and 42% of order interactions. For visualisation, a small number of points with low values were removed. The percentage of points with values lower than the y-axis minimum are as follows for each panel: (a) 1.5%, (b) 0.9%, (c) 0.7%, (d) 11.5%, (e) 8.8%, and (f) 10.6%. Data underlying this figure are given in S10 Data (https://doi.org/10.6084/m9.figshare.12689258.v1). (PDF) [file pbio.3000843.s005.pdf]

included Included Excluded

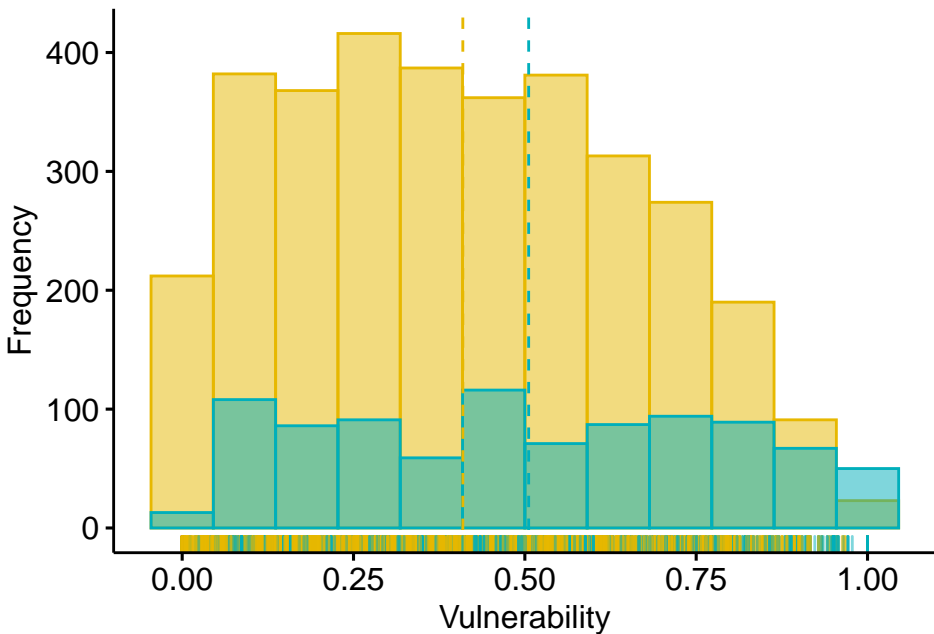

Supplement: S6 Fig — The distribution of vulnerabilities for 3,399 links for which we could analyse feasibility contribution (‘Included’) and 931 links for which we could not analyse feasibility contribution. Vertical dashed lines indicate the mean vulnerability of the 2 groups (0.41 for included links; 0.51 for excluded links). Data underlying this figure are given in S4 Data (https://doi.org/10.6084/m9.figshare.12689258.v1). (PDF) [file pbio.3000843.s006.pdf]
