# Supplementary material for: Estimating the risk of species interaction loss in mutualistic communities
Source: PLoS Biol. 2020 Aug 31;18(8):e3000843. doi: 10.1371/journal.pbio.3000843 (PMC7485972; doi:10.1371/journal.pbio.3000843)
Supplement: S3 Fig — The degree of taxonomic consistency for each interaction at each taxonomic level, for both vulnerability (likelihood of a link being lost) and feasibility contribution (contribution of a link to a network’s feasibility) (ρ = 0.01). Taxonomic consistency is the tendency for properties of an interaction to be more similar across occurrences than expected by chance. Points represent individual interactions. Boxplots represent 5%, 25%, 50%, 75%, and 95% quantiles of the same data, moving from the bottom whisker to the top whisker. Number in bottom left of each panel is the proportion of interactions that exhibited positive consistency (VarianceObserved < VarianceNull). For visualisation, a small number of points with low values were removed. The percentage of points with values lower than the y-axis minimum are as follows for each panel: (a) 1.5%, (b) 1.1%, (d) 7.2%, (e) 6%, and (f) 5.3%. Data underlying this figure are given in S10 Data (https://doi.org/10.6084/m9.figshare.12689258.v1). (PDF) [file pbio.3000843.s003.pdf]

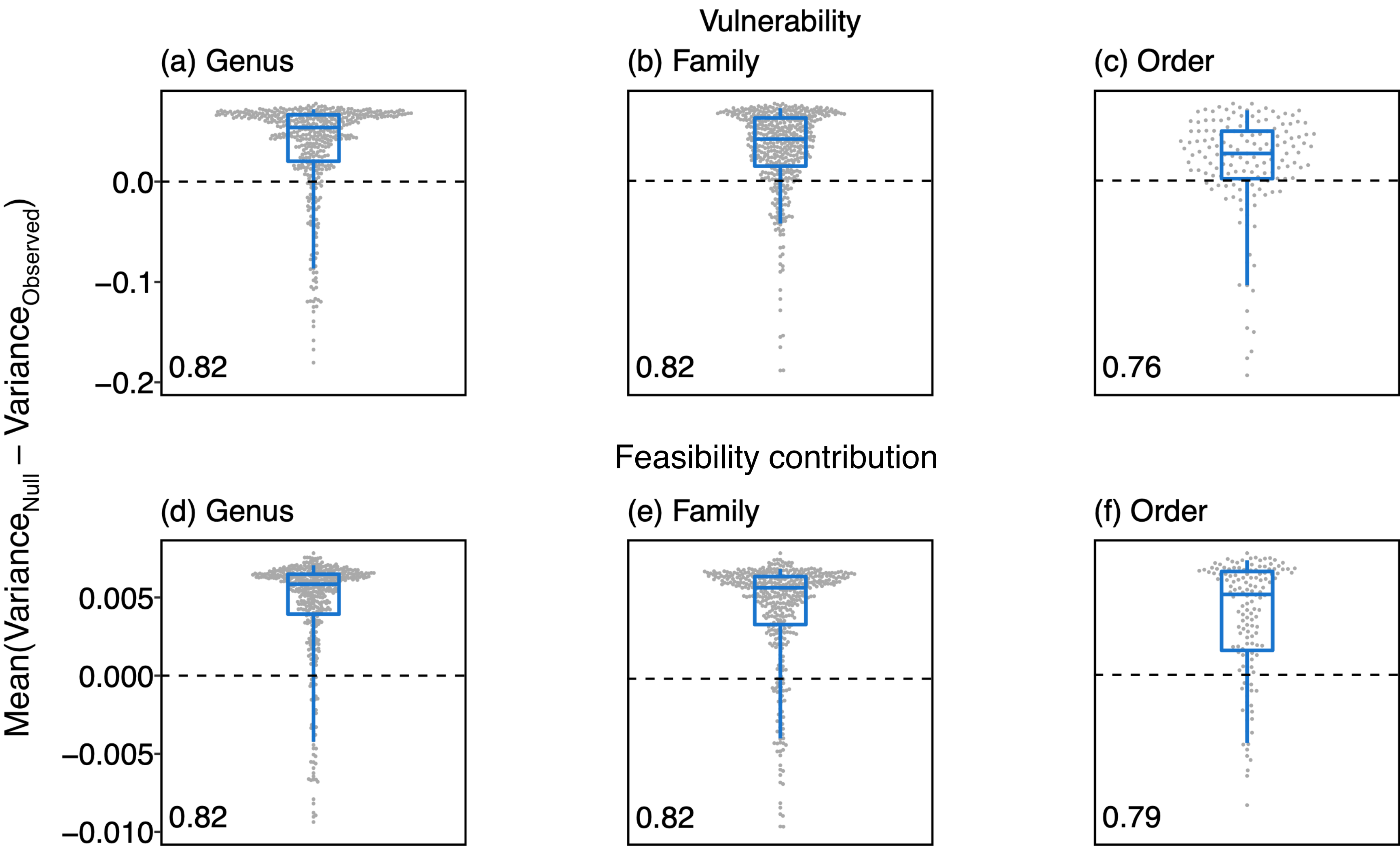
Vulnerability
(a) Genus
(b) Family
(c) Order
0.82
0.82
0.76
Feasibility contribution
(d) Genus
(e) Family
(f) Order
0.82
0.82
0.79
Mean(Variance$_{Null}$ − Variance$_{Observed}$)
0.0
−0.1
−0.2
0.005
0.000
−0.005
−0.010
