# Supplementary material for: Estimating the risk of species interaction loss in mutualistic communities
Source: PLoS Biol. 2020 Aug 31;18(8):e3000843. doi: 10.1371/journal.pbio.3000843 (PMC7485972; doi:10.1371/journal.pbio.3000843)
Supplement: S1 Analysis — (PDF) [file pbio.3000843.s007.pdf]

S1 Analysis: Influence of sampling intensity on vulnerability

We calculated the sampling intensity of each network in our dataset as $\frac{\sqrt{N}}{\sqrt{size}}$, where *N* is the number of interaction events in a network and *size* is the product of the number of plant species and animal species in a network, following [1,2]. We then tested for a correlation between log of a network's sampling intensity and its median vulnerability. We did not find a significant relationship between log of a network's sampling intensity and its median vulnerability (P = 0.0503). While this is approaching significance, the R^2 was low (0.07) and the relationship was driven by a handful of outlier points (identified using Cook's distance with a threshold of 4/n, where n is the sample size). When these were removed, the relationship was even less significant (P = 0.115, R^2 = 0.04).

1. Schleuning M, Fründ J, Klein AM, Abrahamczyk S, Alarcón R, Albrecht M, et al. Specialization of mutualistic interaction networks decreases toward tropical latitudes. Curr Biol. 2012;22: 1925–1931. doi:10.1016/j.cub.2012.08.015
2. Dalsgaard B, Schleuning M, Maruyama PK, Dehling DM, Sonne J, Vizentin-Bugoni J, et al. Opposed latitudinal patterns of network-derived and dietary specialization in avian plant–frugivore interaction systems. Ecography. 2017;40: 1395–1401. doi:10.1111/ecog.02604
