# Supplementary material for: Estimating the risk of species interaction loss in mutualistic communities
Source: PLoS Biol. 2020 Aug 31;18(8):e3000843. doi: 10.1371/journal.pbio.3000843 (PMC7485972; doi:10.1371/journal.pbio.3000843)
Supplement: S2 Analysis — (PDF) [file pbio.3000843.s008.pdf]

S2 Analysis: Analysis assessing the influence of species abundance

It is important to assess the influence of potentially-confounding factors in our analysis, such as abundance. As we do not have independent abundance data for species in our networks, we cannot explicitly test the influence of abundance. Therefore, we have to use total interaction frequency as a proxy for abundance. While this is not always a good proxy [1], and particularly for our analysis there is unavoidable circularity because the vulnerability metric uses interaction frequency in its calculation, it is the best available data to try and address this issue. Additionally, the feasibility contribution metric is independent of interaction frequency.

Due to computational constraints, we carry out our analysis for a representative network close to the median size and connectance of networks in our data: size = 52, connectance = 0.15 (dataset median size = 47, dataset median connectance = 0.16). We generated 100 null networks, using a null model that assumed interaction neutrality by assigning interactions according to a probability matrix, **A**, where element a_{ij} was the relative abundance of animal species i multiplied by the relative abundance of plant species j [1–4]. Therefore, the model assumes that two species with high abundance have a greater likelihood of interacting than two species with low abundance. The model constrained the number of links to be the same as that in the empirical network and ensured that each species had at least one interaction [2]. We used total interaction frequency as a proxy for abundance. This model therefore represents how the network would look if abundance was the only factor governing interactions. If the model can accurately reproduce our results, then we can conclude that our results were driven by abundance. If it does not accurately reproduce our results, then we can conclude that our results were not driven by abundance and other factors must play an important role.

For each of the 100 null networks, we calculated: (i) the mean link vulnerability, (ii) the mean link feasibility contribution, (iii) the standard deviation of the link vulnerability, (iv), the standard deviation of the link feasibility contribution, (v) the slope of the relationship between vulnerability and feasibility contribution. We compared these values to the equivalent values from the empirical version of the network. Results are shown in Table A.

Table A: Comparison of link metrics from an ensemble of null-model-generated networks to empirical values from a representative network with close to the median size and connectance. Null networks were generated using a null model that assumed interaction neutrality.

Property	95% CI	Empirical value
Mean link vulnerability	0.47 – 0.55	0.45
Mean link feasibility contribution	-1.40 – -0.54	-0.003
Standard deviation of link vulnerability	0.27 – 0.32	0.24
Standard deviation of link feasibility contribution	0.067 – 0.093	0.049
Slope of the relationship between vulnerability and feasibility contribution	-0.089 – -0.01	0.08

We found that for none of the metrics did the empirical value overlap the 95% CI of values generated by the null model. Thus, it does not appear that abundance drove the vulnerability or feasibility contribution values, or the relationship between vulnerability and feasibility contribution.

1. Vizentin-Bugoni J, Maruyama PK, Sazima M. Processes entangling interactions in communities: forbidden links are more important than abundance in a hummingbird-plant network. Proc R Soc B Biol Sci. 2014;281: 20132397. doi:10.1098/rspb.2013.2397
2. Vázquez DP, Melián CJ, Williams NM, Blüthgen N, Krasnov BR, Poulin R. Species

abundance and asymmetric interaction strength in ecological networks. Oikos. 2007;116: 1120–1127. doi:10.1111/j.2007.0030-1299.15828.x

3. Maruyama PK, Vizentin-Bugoni J, Oliveira GM, Oliveira PE, Dalsgaard B. Morphological and spatio-temporal mismatches shape a neotropical savanna plant-hummingbird network. Biotropica. 2014;46: 740–747. doi:10.1111/btp.12170
4. Vizentin-Bugoni J, Maruyama PK, Debastiani VJ, Duarte L da S, Dalsgaard B, Sazima M. Influences of sampling effort on detected patterns and structuring processes of a Neotropical plant-hummingbird network. J Anim Ecol. 2016;85: 262–272. doi:10.1111/1365-2656.12459
