# Supplementary material for: Estimating the risk of species interaction loss in mutualistic communities
Source: PLoS Biol. 2020 Aug 31;18(8):e3000843. doi: 10.1371/journal.pbio.3000843 (PMC7485972; doi:10.1371/journal.pbio.3000843)
Supplement: S3 Analysis — (PDF) [file pbio.3000843.s009.pdf]

S3 Analysis: Testing the link generalisation-vulnerability relationship while controlling for local species extinctions

We treat generalised links as more vulnerable than specialised links in our analyses, following an analysis by Aizen et al. [1]. In that analysis, species extinction was not explicitly controlled for as a driver of interaction loss. This is potentially problematic, because we are interested in the drivers of link vulnerability *per se*, rather than as a consequence of partner species going extinct. In other words, link extinction must be separated from species extinction. If this is done, are generalist links still less vulnerable than specialist links? On the one hand it is possible that generalist links may be more vulnerable than specialist links: generalist species may have less fidelity to their partners than specialist species, as they have many other alternative resources, and thus a small change in behaviour could cause a link between two generalists to disappear. On the other hand, many links involve asymmetric specialisation (generalists interacting with specialists) [2,3], which could have lower generalisation than a generalist-generalist link, but still high vulnerability, as the low fidelity of a generalist may cause it to switch away from the specialist. Additionally, specialist species tend to be less abundant [4–7], thus making links between specialists more vulnerable to disruption as the species at low abundance may struggle to search and acquire resources.

It is difficult, then, to settle this issue *a priori*. We therefore re-analysed the original Aizen et al. dataset [8], modifying the analysis to separate link extinction from species extinctions. The Aizen et al. dataset comprises 12 networks from hilltops (sierras) of varying size (from 12 ha to 2147 ha). Aizen et al. use this size gradient as a proxy for habitat loss, and find that the links present in smaller hilltops are nested subsets of the links present in larger hilltops. They go on to find that the links which survive the habitat loss gradient are those which are generalist.

In our re-analysis, we wanted to make sure that link extinctions were not because the partner species are more vulnerable and went locally extinct, but instead because the link itself was more vulnerable. This meant we had to consider links which met the following criteria:

i. The species involved in the focal link must occur in all networks being considered. This removes the possibility of link extinction being a result of species extinction: even if the link is not present, its partner species will be, and so the disappearance of the link will be a consequence of factors other than local-species extinction.

ii. In networks in large hilltops, the species involved in the focal link must interact. This is because the networks in large hilltops are ones where there has not been any habitat loss and thus to determine which interactions are lost along the habitat loss gradient, interactions must occur at the start of the gradient. Given that links in smaller networks are significantly nested subsets of the links in larger networks, many links will meet this criterion.

In networks in small hilltops, species involved in the focal link can either interact or not. This then gives us a set of species pairs where i) the species occur in all networks considered, ii) the species interact in the larger network, and iii) the species either interact or do not interact in the smaller networks. This then allows us to examine each species pair, and to see whether or not their interaction 'survived' the habitat loss from the larger to the smaller networks. We can then determine the characteristics of the links that 'survived' the habitat loss versus those that did not, and test whether the links which survived (i.e. those which were less vulnerable) are those that were more generalist.

Due to our requirement that species pairs must occur in all networks being considered (to remove any effects of species extinction), it was only possibly to conduct our analysis on two networks at a time (one large hilltop, one small hilltop). This is because the more networks that are considered at once, the fewer species pairs occur in all the networks. We therefore considered all possible pairs of large hilltop networks and small hilltop networks. We defined large hilltop networks as those in hilltops greater than 1500ha, while small hilltop networks were defined as those in hilltops less than 600ha. These quantities reflect the distribution of hilltop areas, where a group of networks are clustered in small hilltops, and another group are clustered in large hilltops:

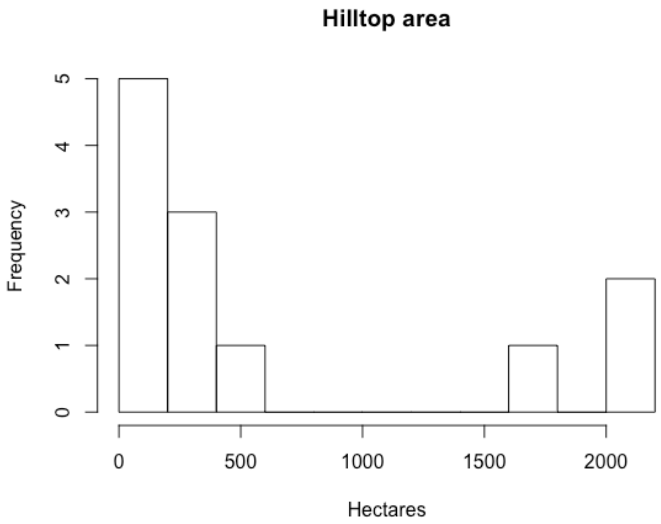

Figure A: Distribution of hilltop sizes among the 12 networks from [1]. Data underlying this figure are given in https://doi.org/10.5061/dryad.cr3ft

For each pair of small and large hilltop networks, we extracted all species pairs which interacted in the large network, and measured the generalisation of all their links. We then considered the subset of these species pairs which also occurred in the small network. We used a one-tailed Wilcoxon signed rank test to test whether the links which survived the transition from the large to small hilltop networks (i.e. species pairs which interacted in both the large and small network) were those that were more generalised in the large network. Conversely, we expected that links that did not survive the transition (i.e. species pairs which interacted in the large network but not in the small network) were those that were more specialist in the large network. The direction of the tests was informed by the hypothesis that generalisation was higher among the set of links that survived the habitat loss (i.e. that generalisation was higher among less vulnerable links). In total there were 27 different combinations of small and large hilltop networks. The analysis could not be completed for three pairs of networks where all species pairs that interacted in the large network, and which also occurred in the small network, also interacted in the small network. In other words, for these three networks, all interactions that met our criteria survived the habitat loss gradient, and thus a comparison of 'survived' *versus* 'not survived' links was not possible.

We found that generalisation was significantly higher for links which survived the habitat loss (less vulnerable links) in 79% of network pairs (19/24). Thus, links which were generalist in the large network tended to survive the habitat loss, while specialist links tended to not survive. In other words, vulnerable links were those that were significantly more specialised (had significantly lower generalisation). Note that if we count the 3 networks for which tests could

not be completed as being cases where generalisation was not significantly higher for less vulnerable links, then our hypothesis was true in 70% of network pairs (19/27). We also note that in 96% of network pairs (23/24), mean link generalisation was higher for surviving (less vulnerable) interactions than vulnerable interactions, suggesting that, in these data, there is little support for the hypothesis that generalised links are more vulnerable.

Due to the multiple tests we conduct, there is an increased probability of Type I errors. Therefore, we used the equation of [9] to calculate the likelihood of obtaining the number of significant tests that we did. The probability of a given number of significant tests, p , is given by the equation

$$p = [N!/(N - K)!K!] \times \alpha^{K}(1 - \alpha)^{N - K},$$

where N is the number of tests conducted, and K is the number of tests below the significance level α. We found that the probability of obtaining our results by chance was 6.27×10^{-21}. Therefore, despite the possibility of Type I errors from multiple tests, it was highly unlikely that our results were by chance. From this we conclude that our original definition of vulnerable links being more specialised remains true, even when controlling for species extinctions.

1. Aizen MA, Sabatino M, Tylianakis JM. Specialization and rarity predict nonrandom loss of interactions from mutualist networks. Science. 2012;335: 1486–1489. doi:10.1126/science.1215320
2. Bascompte J, Jordano P, Olesen JM. Asymmetric coevolutionary networks facilitate biodiversity maintenance. Science. 2006;312: 431–433. doi:10.1126/science.1123412
3. Vázquez DP, Aizen MA. Asymmetric specialization: A pervasive feature of plant-pollinator interactions. Ecology. 2004;85: 1251–1257. doi:10.1890/03-3112
4. Olesen JM, Bascompte J, Elberling H, Jordano P. Temporal dynamics in a pollination network. Ecology. 2008;89: 1573–1582. doi:10.1890/07-0451.1
5. Vázquez DP, Aizen MA. Null model analyses of specialization in plant-pollinator interactions. Ecology. 2003;84: 2493–2501. doi:10.1890/02-0587
6. Simmons BI, Vizentin-Bugoni J, Maruyama PK, Cotton PA, Marín-Gómez OH, Lara C, et al. Abundance drives broad patterns of generalisation in plant–hummingbird pollination networks. Oikos. 2019;128: 1287–1295. doi:10.1111/oik.06104
7. Dupont YL, Hansen DM, Olesen JM. Structure of a plant--flower-visitor network in the high-altitude sub-alpine desert of Tenerife, Canary Islands. Ecography. 2003;26: 301–310.
8. Gilarranz L, Sabatino M, Aizen M, Bascompte J. Data from: Hotspots of mutualistic networks. Dryad, Dataset; 2015. doi:https://doi.org/10.5061/dryad.cr3ft
9. Moran MD. Arguments for rejecting the sequential bonferroni in ecological studies. Oikos. 2003. pp. 403–405. doi:10.1034/j.1600-0706.2003.12010.x
