# Supplementary material for: Estimating the risk of species interaction loss in mutualistic communities
Source: PLoS Biol. 2020 Aug 31;18(8):e3000843. doi: 10.1371/journal.pbio.3000843 (PMC7485972; doi:10.1371/journal.pbio.3000843)
Supplement: S4 Analysis — (PDF) [file pbio.3000843.s010.pdf]

S4 Analysis: Identifying the domain of δ values where vulnerable links have the highest feasibility contribution

δ is the mutualistic trade-off parameter [1]. It is part of the mutualistic benefit equation in the dynamics model used to calculate link feasibility contribution. In the model, mutualistic benefit $\gamma_{ij} = \gamma_0 L_{ij}/d_i^{\delta}$, where $L_{ij} = 1$ if there is a link between species i and j and zero if there is no link; d_i is the degree of species i; δ is the mutualistic trade-off; and γ_0 is the overall level of mutualistic strength. δ "modulates the extent to which a species that interacts with few other species does it strongly, whereas a species that interacts with many partners does it weakly" [2].

When $\delta = 0$, the mutualistic benefit between species i and j is the same, regardless of the degree of species i. In other words, a species that interacts with few other species does so equally as strongly as a species that interacts with many other species. Conversely, when $\delta = 1$, the mutualistic benefit between species i and j decreases as the degree of species i increases. Thus, a species that interacts with few other species does so strongly, whereas a species that interacts with many other species does so weakly. This is demonstrated in the below figure, assuming $L_{ij} = 1$ and $\gamma_0 = 1$.

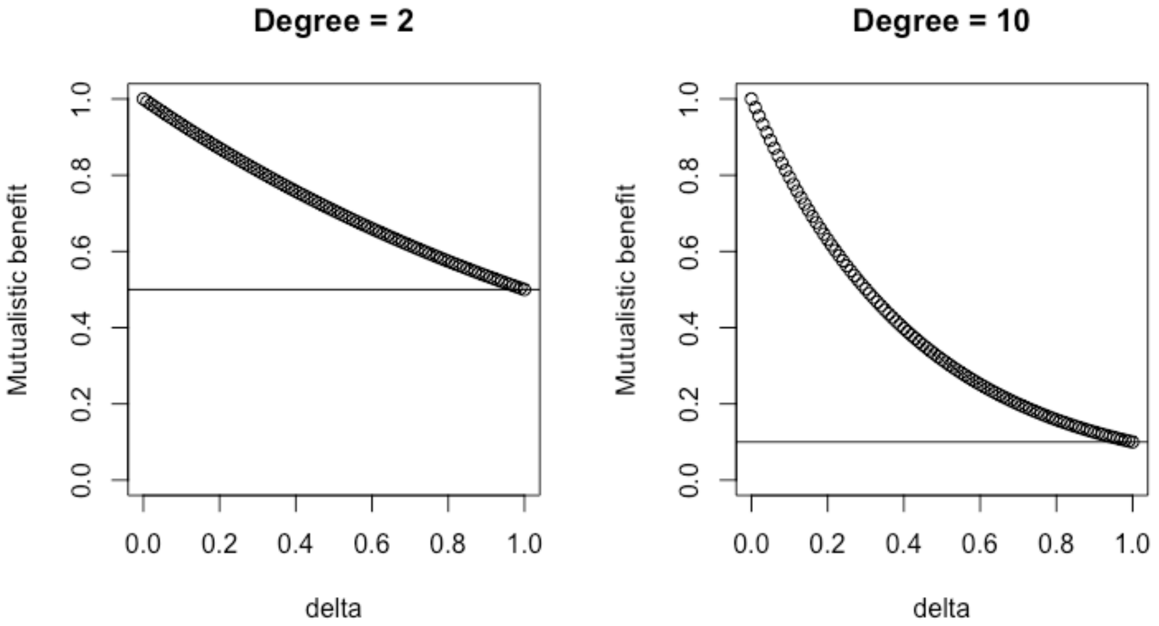

Figure A: The relationship between δ and the mutualistic benefit between species i and j. The left panel shows a case where species i has a degree of 2; the right panel shows a case where species j has a degree of 10. When $\delta = 0$, the mutualistic benefit between i and j is the same regardless of the degree of i. When $\delta = 1$, the mutualistic benefit between i and j decreases with degree: it is lower for the case where i has a degree of 10, than the case where it has a degree of 2. Data underlying this figure are given in S5 Data (https://doi.org/10.6084/m9.figshare.12689258.v1).

As can be seen, when $\delta = 0$, both the degree = 2 and the degree = 10 species i have the same mutualistic benefit with species j. However, when $\delta = 1$, the mutualistic benefit decreases to 0.5 in the degree = 2 case and 0.1 in the degree = 10 case, as indicated by the horizontal grey lines.

δ is an important parameter that could influence the observed relationship between vulnerability and feasibility contribution. It is therefore necessary to identify the range of δ values in which vulnerable links have the highest feasibility contribution (i.e. the range of δ values for which our conclusion holds). We have therefore repeated our analysis at different values of δ to identify the domain of δ values in which vulnerable interactions have the highest feasibility contribution. Since vulnerability requires interaction frequency for its calculation, which in turn ought to determine δ (which is now varied for this analysis), we here examine how varying δ affects the relationship between *generalisation* and feasibility contribution. We additionally run our analyses for three levels of average mutualistic strength, to understand how our results are affected by changes in this parameter. We repeated our analysis for average mutualistic strength set to 0.1, 0.5 and 0.9 of the average mutualistic strength at the stability threshold.

As expected, the δ parameter influences the slope of the relationship between generalisation and feasibility contribution, with qualitatively similar results for both zero ($\rho = 0$) and weak ($\rho = 0.01$) competition. Only for high δ values (>~0.6-0.7), the slope between generalisation and feasibility contribution is positive, whereas for most δ values the slope is negative i.e. for most δ values, more vulnerable (less generalised) links have the highest feasibility contribution. The vertical black line indicates the value of the empirically-estimated δ value that was used in the main text. At the empirical delta value, the slope is negative at all mutualistic strengths; thus, our results are not sensitive to mutualistic strength in terms of directionality of the relationship between generalisation and feasibility contribution i.e. at the empirical δ value, at all mutualistic strengths tested, more vulnerable (less generalised) links have the highest feasibility contribution.

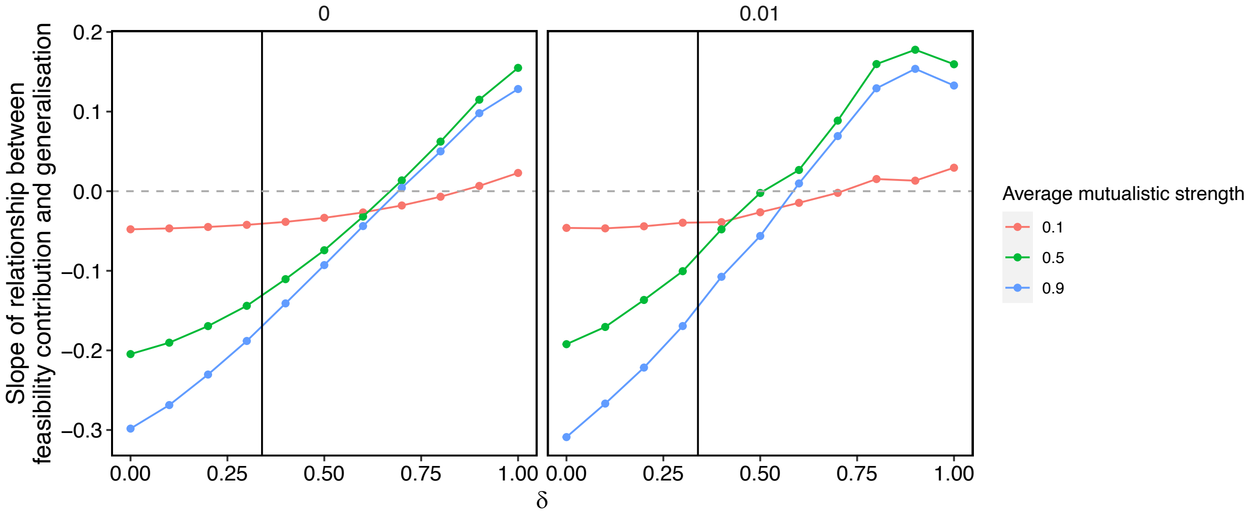

Figure B: Relationship between δ and the slope of the relationship between feasibility contribution and vulnerability. Results are shown both zero (Left: $\rho = 0$) and weak (Right: $\rho = 0.01$) competition. Each line refers to a different average mutualistic strength. The value of 0.1, 0.5 or 0.9 means that the average mutualistic strength was set to 0.1, 0.5 or 0.9 of the average mutualistic strength at the stability threshold, respectively. Data underlying this figure are given in S6 Data (https://doi.org/10.6084/m9.figshare.12689258.v1).

Note that the vulnerability metric has an opposite direction to generalisation: more generalised links are less vulnerable and specialist links are more vulnerable. Thus, the positive slope between vulnerability and feasibility contribution presented in the main text is equivalent to a negative slope between generalisation and feasibility contribution in this analysis.

Application to random networks

To test whether our results are specific to empirical networks or would also hold for a random bipartite network, we repeated the above analysis for an ensemble of random bipartite networks with degree distributions comparable to those in empirical data. We chose to generate random networks that had a size and connectance the same as the median size and connectance of networks in our dataset (47 species, 0.16 connectance). We thus used the 'curveball' algorithm [3] to produce 50 randomisations of the network from our dataset which had this median size and connectance. The curveball algorithm randomises the network structure while maintaining the degree distribution [3]. The results are shown below

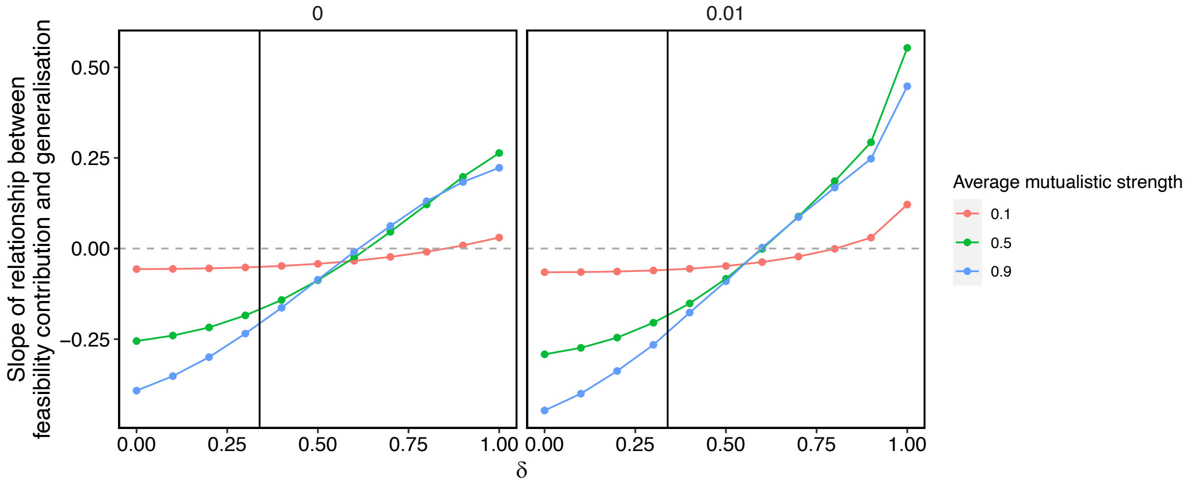

Figure C: Relationship between δ and the slope of the relationship between feasibility contribution and vulnerability for an ensemble of 50 randomisations of the network with median size and connectance from our dataset. Randomisations constrained degree distribution. Results are shown both zero (Left: $\rho = 0$) and weak (Right: $\rho = 0.01$) competition. Each line refers to a different average mutualistic strength. The value of 0.1, 0.5 or 0.9 means that the average mutualistic strength was set to 0.1, 0.5 or 0.9 of the average mutualistic strength at the stability threshold, respectively. Data underlying this figure are given in S7 Data (https://doi.org/10.6084/m9.figshare.12689258.v1).

These results from the randomisations of the median network are qualitatively the same, but quantitatively different, to the results from across all empirical networks (Figure B). While these analyses are not directly comparable, because they involve different sets of networks, this result implies that the broad pattern of how the slope between generalisation and feasibility contribution varies with delta and mutualistic strength is heavily constrained by empirical degree distributions. However, particular features of empirical networks beyond the degree distribution may determine the exact quantitative values of these relationships. Overall, this analysis confirms that our result is robust for bipartite networks beyond our dataset that resemble the structure of empirical mutualistic communities.

1. Saavedra S, Rohr RP, Dakos V, Bascompte J. Estimating the tolerance of species to the effects of global environmental change. Nat Commun. 2013;4: 2350. doi:10.1038/ncomms3350
2. Rohr RP, Saavedra S, Bascompte J. On the structural stability of mutualistic systems. Science. 2014;345: 1253497–1253497. doi:10.1126/science.1253497
3. Strona G, Nappo D, Boccacci F, Fattorini S, San-Miguel-Ayanz J. A fast and unbiased procedure to randomize ecological binary matrices with fixed row and column totals.

Nat Commun. 2014;5: 4114. doi:10.1038/ncomms5114
