# Supplementary material for: Estimating the risk of species interaction loss in mutualistic communities
Source: PLoS Biol. 2020 Aug 31;18(8):e3000843. doi: 10.1371/journal.pbio.3000843 (PMC7485972; doi:10.1371/journal.pbio.3000843)
Supplement: S5 Analysis — (PDF) [file pbio.3000843.s011.pdf]

S5 Analysis: Is visitation frequency a good proxy for abundance?

As our analysis uses frequency as an explanatory variable, it is important to understand to what extent visitation frequency is influenced by, or independent from, abundance. We attempted to look at this question using an updated version of a hummingbird-plant pollination network from [1], which comes with independent abundance data. The updated data were from [2–4].

We correlated the observed visitation frequency with the visitation frequency that would be expected from abundance alone. To generate the abundance-driven frequencies, we followed the standard technique of [5], and created a probability matrix where the probability of each species interacting equalled the product of their relative abundances. Thus, abundant species are more likely to interact than rare species pairs. We then used a negative binomial model to correlate observed visitation frequency of each species pair against the corresponding probability of interaction that would be expected from abundance alone.

We found a significant ($P = 0.16$), but very weak (McFadden Pseudo-$R^2 = 0.01$), correlation between abundance-driven probability of interaction and observed interaction frequency (Figure A 'Empirical' panel).

To test whether this weak relationship was a peculiarity of our data, we repeated the analysis, using a range of idealised abundance distributions instead of the empirical abundance distribution. Species were assigned an abundance from each distribution based on their percentile. For example, a species in the 88th percentile in the empirical abundance data was assigned the corresponding 88th percentile value from the idealised distribution. This process was repeated separately for plants and pollinators. We considered four possible abundance distributions: exponential, Fisher log series, uniform and lognormal. The exponential distribution had a rate of 1. The Fisher log series requires parameters for the number of species S, the total number of individuals N, and two measures which control the shape of the distribution: a measure of biodiversity, α, and the Fisher parameter y [6]. Following [6], we set S equal to the number of species in the focal trophic level in the network, and $y = 0.5$ (the value of y does not matter as we later convert to relative abundances). Using these values, we solved for α and N. The lognormal had a mean equal to the mean of the empirical abundances, and a standard deviation of 1. All idealised abundances were converted to relative abundances.

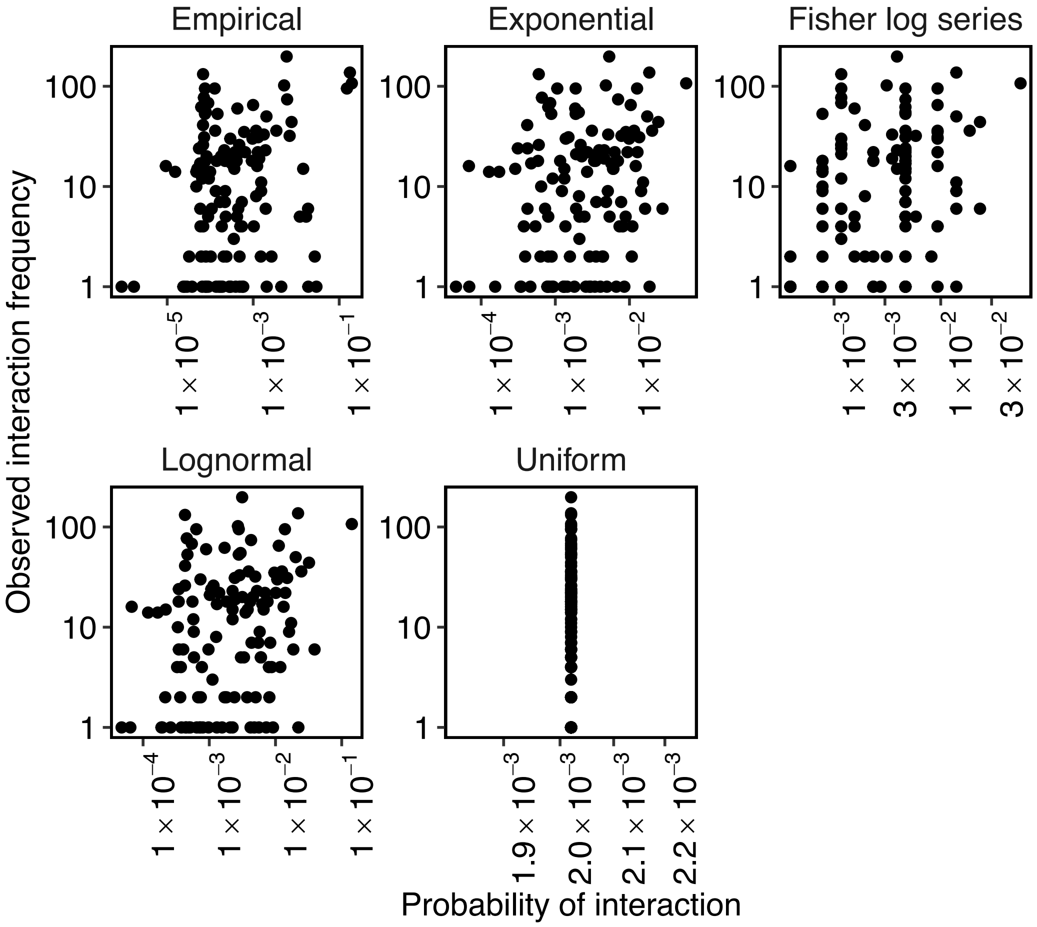

Figure A: Relationships between observed interaction frequency and the probability of interaction derived from empirical, or idealised, abundance distributions. Data underlying this figure are given in S8 Data (https://doi.org/10.6084/m9.figshare.12689258.v1).

We found similar results for the idealised abundances as we did for the empirical abundances. Fisher log series and exponential abundance-driven interaction probabilities were significantly related to empirical interaction frequency (P = 0.03 and 0.03 respectively), but for lognormal and uniform no significant relationship was found. For the two significant relationships, McFadden pseudo-R^2 remained very low: 0.006 for exponential and 0.006 for Fisher log series.

These results suggest that abundance may be a poorer predictor of visitation frequency than traditionally thought, suggesting that our correlation between vulnerability and feasibility might not be biased by the visitation frequency uncorrected for abundances.

1. Vizentin-Bugoni J, Maruyama PK, Sazima M. Processes entangling interactions in communities: forbidden links are more important than abundance in a hummingbird-plant network. Proc R Soc B Biol Sci. 2014;281: 20132397. doi:10.1098/rspb.2013.2397
2. Sonne J, Vizentin-Bugoni J, Maruyama PK, Araujo AC, Chávez-González E, Coelho AG, et al. Ecological mechanisms explaining interactions within plant-hummingbird networks: morphological matching increases towards lower latitudes. Proc R Soc B Biol Sci. 2020;287: 20192873. doi:10.1098/rspb.2019.2873
3. Sonne J, Vizentin-Bugoni J, Maruyama PK, Araujo AC, Chávez-González E, Coelho AG, et al. Ecological mechanisms explaining interactions within plant-hummingbird

networks: morphological matching increases towards lower latitudes, v5, Dryad, Dataset. 2020. doi:https://doi.org/10.5061/dryad.dncjsxkw2
4. Vizentin-Bugoni J, Maruyama PK, Debastiani VJ, Duarte L da S, Dalsgaard B, Sazima M. Influences of sampling effort on detected patterns and structuring processes of a Neotropical plant-hummingbird network. J Anim Ecol. 2016;85: 262–272. doi:10.1111/1365-2656.12459
5. Vázquez DP, Chacoff NP, Cagnolo L. Evaluating multiple determinants of the structure of plant-animal mutualistic networks. Ecology. 2009;90: 2039–2046. doi:10.1890/08-1837.1
6. de Aguiar MAM, Newman EA, Pires MM, Yeakel JD, Boettiger C, Burkle LA, et al. Revealing biases in the sampling of ecological interaction networks. PeerJ. 2019;2019: e7566. doi:10.7717/peerj.7566
