# Supplementary material for: Estimating the risk of species interaction loss in mutualistic communities
Source: PLoS Biol. 2020 Aug 31;18(8):e3000843. doi: 10.1371/journal.pbio.3000843 (PMC7485972; doi:10.1371/journal.pbio.3000843)
Supplement: Attachment — Submitted filename: ResponseToReviewers_r1.pdf [file pbio.3000843.s013.pdf]

[*Authors note: line numbers in this response to reviewers refer to the clean version of the manuscript without track changes***]**

Editor:
The rationale behind this decision is that although the reviewers are positive about the questions you're asking, they raise significant concerns about some of your definitions, the potentially circular reasoning (see revs #1 and #3), and the lack of methodological clarity. We think that addressing some of the graver concerns of the reviewers would require extensive additional work to achieve the level needed for PLOS Biology, and that some of the current claims may not survive the revisions.

1. Response: We thank the Editor for giving us the chance to revise and resubmit our manuscript. We have extensively revised our manuscript and addressed all the Reviewers concerns below, in particular by improving definitions, explaining that our analysis is not circular, and significantly improving the clarity of our method descriptions.

Reviewer #1:
[identifies himself as Jean-Francois Arnoldi]

The manuscript by Simmons et al is an analysis of empirical pair-wise mutualistic plant-animal networks (pollination or seed dispersal), and the ecological risk posed by interaction loss.

The authors propose that the risk associated to a given interaction should encode the likelihood of loosing the link (vulnerability) as well as the importance of the link, that is: the impact that its loss will have on the ecosystem (e.g. reduction of function provisioning and/or biodiversity loss).
Specifically, the authors focus on biodiversity by measuring the importance of links for species coexistence.

The authors find that the most vulnerable links (most likely to be lost) also tend to be the most important for coexistence. Vulnerability is computed directly from data, and encompasses frequency of the interaction (e.g number of visits of a pollinator per day); as well as the degree of generalism of the species involved in the interaction (how many links they rely on). Importance for coexistence, on the other hand, is quantified as feasibility via a Lotka-Volterra model parametrisation. Feasibility is quantified as the intensity of environmental perturbation required to push at least one species to extinction (cf. the work of Saavedra et al, cited profusely in the MS).

The authors also find that vulnerability and importance of a given link between species are roughly conserved across data sets, thus suggesting that such characteristics are 'intrinsic' properties of the species pair. The authors naturally conclude that vulnerable links should be the focus of conservation efforts across systems.

Assessment:

Overall the manuscript is very well written, the topic highly relevant and the questions well posed. The ecological interpretations make sense, and follow the results.

2. Response: We thank the reviewer for their positive comments on our manuscript.

My critique is basic, yet potentially severe: I fear that the method is circular. Indeed, it seems to me that the relationship between vulnerability and importance is inherent to the way these notions are defined. If species must have links to persist; if the measure of vulnerability of a link between species is a function of the number of links that those species rely on, it is not surprising that a vulnerable link is also important. For instance, if the link between species A and B is the only link that A and B have, this implies high vulnerability. Loosing this link will imply the loss of species A and B, since it is the only link they rely on (high importance). The relationship between vulnerability and importance is thus present by definition.

Instead, I would suggest to study the link between generalism and feasibility which, if I am right, should be quite strong. This would provide additional evidence (although purely theoretical) that generalism of mutualistic links promotes coexistence. Then I would study the link between vulnerability (but now measured only via the empirical frequency of occurrence) and generalism. Unfortunately, in this case I doubt that it will be possible to say that "more vulnerable links are also the most important". But it would still allow the authors to exhibit those interactions critical for coexistence.

3. Response: We thank the Reviewer for raising this point. However, we do not believe the method is circular. At first sight it is reasonable to think that the correlation between vulnerability and importance is present by definition, but it is not true in our case as we explain below.

First, the example used by the Reviewer neglects that vulnerability is a metric comprising both link generalisation and frequency. Thus, "if the link between species A and B is the only link that A and B have, this implies high vulnerability", is only true when link frequency (visitation rate in our study) is low. In our datasets we did not find a strong relationship between generalisation and frequency. While the correlation was positive and significant, it was noisy (see Supplementary figure S2) and frequency explained only 8.9% of the variance in generalisation.

Second, if the link between species A and B is the only link that A and B have, then by definition these species are not connected to the rest of the network, and exist in isolation in a small two-species network. In our study, all our networks form a single connected component, and so we do not have any situations where two species exist in isolation.

Third, "Losing this link will imply the loss of species A and B, since it is the only link they rely on (high importance)" is an example specific to a case where, following link removal, both partner species have no links. We do not consider any cases where removing a link results in one or both partners having no links, because feasibility cannot be calculated when a species has no links; see also response #10 below, L431-443 and Supplementary Figure S6 for a discussion of the implications of this constraint. Additionally, the extinction of A and B

following the removal of their only link is what to expect if we would have measured persistence, where simulations are used to see whether species go extinct in the presence of perturbations. In a persistence framework, we agree that deleting a link between two specialist species is likely to increase their chance of extinction. However, here we do not consider whether species are lost following link removal; instead, we consider feasibility, which is the range of conditions under which all species in the network can coexist. Thus if two species lose their link, the question is not whether those species will be lost, but whether the loss of this link results in a larger or smaller range of conditions under which all species in the network (including the ones to which the link belongs to) can survive. Thus, removing a link of a species could lead to the extinction of that species in a persistence framework, but the overall effect on feasibility in the remaining community may increase or decrease and this is not so obvious to deduce. Indeed, no explicit relationship between persistence and feasibility is to be expected (pers comm R Rohr). Thus it is not easy to conclude, in a feasibility framework, that species who have their link removed will decline to extinction, even if they have few other interactions.

But perhaps the clearest evidence that the relationship between vulnerability and importance is not to be expected by definition can be found in our correlations (Fig 2 main text). While we do find an interesting positive correlation between importance and vulnerability, the correlation is not so strong as to be expected by definition. Instead, there is noise in the relationship e.g. there are low vulnerability links which are important, and high vulnerability links that are not important. This is also true when only considering the relationship between generalisation and importance: there are specialised links which are not important (and even cases where removing a specialised link *increases* the feasibility of the network i.e. has negative importance), and highly-generalised links that are important. We now include this relationship in the supplementary information following the reviewer's suggestion (Figure S2). Such patterns would not arise if the correlation was present by definition. We may have found that frequency and generalisation of links are good predictors, but we have not discovered a perfect explanation of link importance. Our model still leaves 40% of variance in importance unexplained by either vulnerability or the network identity random effect.

Thus, we do not believe the correlations we present are so strong as to suggest a trivial relationship that arises purely from circularity or which could be derived analytically. Instead, we believe we have uncovered useful correlates of link importance in mutualistic networks, but with unexplained variance that is likely accounted for by factors (topological or otherwise) that future research will hopefully uncover.

We, however, absolutely agree the issues raised by the reviewer are important to explain and have partly arisen due to a lack of clarity in the methodology (also raised by reviewer 3). We have therefore added a section to the main text addressing the apparent circularity between importance and vulnerability (L221-225). We also describe the feasibility metric in more detail, alongside an expanded justification of why we chose it (L369-443). We have also followed the reviewer's suggestion and we have added plots of the relationship between generalisation and importance and frequency and generalisation to the supplementary material (Figure S2).

Finally, on the result concerning the conservatism of vulnerability, I would need a more detailed explanation of the method to make a fair assessment. It seems that the authors are saying that the links are not completely randomly redistributed between species across systems, which is not too surprising. But I could be wrong in my interpretation.

4. Response: We agree with the Reviewer that this method could be more clearly explained. We have therefore completely rewritten, and substantially expanded, our description of the methodology of this analysis to significantly improve clarity and detail (L451-527). As part of this, we have included a new explanatory figure (Figure 4) to help communicate the steps of the analysis to readers.

Reviewer #2:
[identifies himself as Shawn J. Leroux]

Summary:
Ecological communities are changing in response to global stressors such as land-use and climate change. Studying and predicting community change is an active field of research but most of this research focuses on species dynamics. Recent research shows that species interactions are more sensitive to change than species, therefore, we need to better understand the dynamics of these interactions. In this study, the authors analyze a large data set of mutualistic communities to determine the risk of species interaction loss on community persistence. They develop novel metrics to measure the vulnerability and importance of species interactions and show that the most vulnerable interactions may also be the most important for community persistence. The metrics and findings presented here provide guidelines for the conservation of species interactions under global change.

General comments:
1. This is a very thought provoking and original manuscript. It really challenged me to think about how to measure the vulnerability and importance of species interactions and I think it represents a major step forward in our approach to tackling this problem. Congratulations on a very nice study!

5. Response: We thank the reviewer for their kind comments about our study.

2. A major part of the novelty is in the integration of the metrics of link vulnerability and link importance. Yet, some of the details on these metrics were unclear. Specifically:
a. I appreciate the author's definition of interaction and link (lines 80-81). Am I right that interactions are defined at the metaweb level and link at the local web level? If so, then I recommend the authors infuse this language here as it is common currency in network analyses.

6. Response: We agree with the Reviewer that our definition of these terms was unclear. We have now reworded the definition to include the 'metaweb' and 'local' web terminology suggested by the Reviewer, and have added a much clearer example to explain the terms (L71-78).

b. The measure of vulnerability is taking the Aiken approach a step further. I think there is reasonable support to suggest that weak links between specialist partners are more vulnerable than strong links between generalists. But this measure of vulnerability also contrasts with some more common measures – particularly in studies of community sensitivity to node (species) extinctions. I recommend the authors add some text to explain how their measure relates to more common measures of vulnerability such as body size (larger more vulnerable), trophic level (higher more vulnerable) etc. I understand that these measures are usually more node-based but I think if the authors want others to adopt their approach, then the onus is on them to demonstrate how their approach a) compares/differs from other approaches and b) provides novel insights. A specific example of vulnerability in a conservation context may also help to communicate the ecological interpretation of this metric.

7. Response: We thank the Reviewer for raising this point, and we agree that ours is unlikely to be the only sensible metric of link vulnerability. The two measures the Reviewer cites (bodymass and trophic level) are mostly used as vulnerability measures in unipartite food webs, rather than the bipartite plant-animal mutualistic networks we use here. We find it hard to translate bodymass and trophic level as meaningful and measurable quantities for plant and pollinator species. Because bipartite networks comprise only two levels of nodes (with all plants in one level and all pollinators in the other level), trophic level as measure of link vulnerability would not be informative in this case. Similarly, bodymass is not widely used in pollinator or seed disperser networks to indicate vulnerability, especially for pollinator networks, as bodymass data is not usually available for all the insect species in a network. Additionally, because all links in our networks are between an animal and a plant, measuring the vulnerability of a link as, for example, the mean bodymasses of the two species involved in a link, would require detailed data on plant biomass at each fieldsite which is not available.

Nonetheless, we could imagine other measures related to species traits, rarity or life-strategies that could be developed in combination as metrics of vulnerability. To a certain extent, link frequency (measured as visitation frequency) takes partly such species properties into account as the most abundant species, or species with longer phenologies, might have higher visitation rates.

However as the Reviewer says, all species measures are node-based vulnerability metrics, which do not necessarily translate into link vulnerability. Aizen et al (Science 2012, 335, 1486–1489) provide the only vulnerability metric for links in mutualistic networks that we know of, and it is empirically validated, so we feel this is a strong case to use it over other possible measures. Additionally, Aizen et al.'s metrics encompass reasons for link loss beyond species extinction. For example, more specialised links are more vulnerable, not necessarily just because the species involved may be more vulnerable, but also because the links cannot be 'subsidized' or 'rescued' by other species if the interacting species become isolated in space or time while remaining extant (Aizen et al Science 2012, 335, 1486–1489). Link vulnerability metrics based purely on measures of node vulnerability may not capture this possibility, and instead might represent link loss solely as a function of the likelihood of species loss.

Triggered by the above, we have now added a discussion to the text where we justify the choice of the Aizen metric by comparing it to other approaches based on node vulnerability; and encourage future experimental studies to find other possible correlates of link vulnerability (L296-305, and L354-365). We also mention that bodymass and trophic level could be good candidates for link vulnerability measures in unipartite networks, such as food webs (L298-299).

We also agree an example of our vulnerability measure being used would help communicate the ecological interpretation of the metric. We now added a description of the experiment by Aizen et al where they show that link generalisation and frequency correlate with link vulnerability to habitat loss (inferred from examining the link composition of communities along a gradient of sierras of different sizes to simulate habitat loss) (L121-127). We agree this will help ground the metric in a clear example and should improve its ecological interpretation by readers.

c. I am confused about the nature of the interaction data. Aizen used weighted interactions (i.e., # of times an individual visited a flower). In lines 286-290, it seems like this study uses binary data and re-interprets the Aizen frequency metric in terms of # of species visiting instead of # of individuals visiting? I could be wrong but something does not match here so I would appreciate further details.

8. Response: We use weighted interactions as in Aizen. Frequency in our analysis is defined as stated by the Reviewer: # of times an individual visited a flower. We have improved the text on L328-330 and L345 to clarify that we are using weighted data based on individuals visiting, not binary data.

d. I really like the adoption of the Song feasibility domain metric but as above I want more details. I think the steps of this analysis can be better communicated with an analysis pipeline diagram. The authors outline how they define key parameters in the simulations but they do not bring the reader back to how the material on lines 320-340 result in feasibility measures.

9. Response: We agree that the links between the dynamic model and our feasibility measure were not as clear as they could be in the original text. Rather than use a pipeline diagram, we have significantly expanded and restructured this section of the methods to make it clear how the material on lines 320-340 in the original manuscript result in feasibility measures (L368-449, particularly L419-443).

Also, what are the implications of not measuring feasibility for 931 links? Are these links the most/least vulnerable ones? A bit more information on these links would be useful to help the reader interpret them.

10. Response: We thank the Reviewer for raising this point and we agree it is useful to provide some information on the 931 excluded links. We found that the vulnerability of these links was slightly higher than the 3399 links for which we could analyse feasibility (mean vulnerability of 0.41 versus 0.51 for included and excluded links respectively). If we were

able to calculate feasibility for all links, and had a complete dataset of 4300 links, the mean vulnerability would be 0.43. So the mean vulnerability of the set of links we analysed (0.41) is not too different from this. Additionally, from examining histograms of the two sets of links, our analysed set covers the full span of vulnerability values fairly well, with the most notable difference being a higher proportion of links with higher vulnerabilities in the '931 set' (which is what causes the difference in mean mentioned above). Thus, we conclude there is a slight difference in the distribution of the two sets, but we do not think this will significantly affect our conclusions as the two distributions are similar..

We agree it is important to make readers aware of all this, and so we have added the histogram to the supplementary information (Figure S6) and referenced this in the main text with some discussion (L431-443).

3. I appreciate the author's desire to inform conservation of species interactions but I recommend the authors be a bit more careful about their scope of inference. For example, on line 106-108 – the specific metric of vulnerability used here does not have anything to do with the presence of a stressor like habitat loss. Similar comment for lines 124-126 (and 209-211) – the authors have not demonstrated that vulnerable links are most likely to be lost in face of environmental changes. They assume that vulnerable links are most sensitive and indeed this is critical to make an argument that the metric of vulnerability here is useful. Other studies may measure the relationship between vulnerability and e.g., habitat loss (Aizen) but as measured in this study, vulnerability is not related to environmental change.

11. Response: Our reason for mentioning habitat loss as a possible driver of the loss of vulnerable links in L106-108 (in the original text) was motivated by the work of Aizen (Science 2012, 335, 1486–1489) that explicitly related the two components of vulnerability (link frequency and generalisation) to habitat loss in an experimental setting. However, we agree that Aizen et al related link frequency and generalisation separately to habitat loss, while we combine them into a novel index of vulnerability which itself has not been explicitly related to habitat loss. We therefore have removed mention of our metric capturing the response to habitat loss in and rephrased the sentence to state that our vulnerability metric *aimed* to capture vulnerability to a stressor, but not that it explicitly does (L119-121).

For L124-126 (in the original text) we have significantly softened the language. Before we stated that the importance-vulnerability positive correlation indicated that links which contribute most to the feasibility of our communities are those links that are most likely to be lost in the face of environmental changes. We now state that we find a correlation between importance and vulnerability values and that this *may* indicate these links are likely to be lost in the presence of environmental change (L148-151).

L209-211 (in the original text) are part of the following section: "The positive relationship between vulnerability and importance means that the more vulnerable a link is, the more likely it is to have a negative impact on network feasibility if it is lost. Vulnerability is therefore an important indicator of the extent to which a link supports or hinders a community's ability to tolerate environment variation and, thus, species' long-term persistence". Here we are not claiming that vulnerable links are most likely to be lost in face of environmental changes as

stated by the Reviewer; instead we are talking about how vulnerability is related to feasibility, and how feasibility is "an important indicator of the extent to which a link supports or hinders a community's ability to tolerate environment variation". Feasibility is widely referred to in terms of the amount of environmental variation that can be tolerated without any species extinction in a community and so we use this terminology here. See e.g. Song et al 2018. A guideline to study the feasibility domain of multi-trophic and changing ecological communities. Journal of Theoretical Biology, 450, pp.30-36.

4. I also think the taxonomic consistency analysis is very interesting and useful. But I could not follow how consistency in both vulnerability and importance relates to consistency in only vulnerability or only importance. The levels of consistency are very different in these cases and I would appreciate some discussion on this.

12. Response: We thank the Reviewer for their positive comments about the taxonomic consistency analysis. We do not relate consistency in both vulnerability and importance to consistency in only vulnerability or only importance, and therefore think this a confusion that results from our unclear wording, which referred to 'consistency for both vulnerability and importance'. We have revised the wording in this section so that the results are more clearly expressed (L183-195, L197-199). Additionally, in response to comments from Reviewer 1, we have completely rewritten, and substantially expanded, our description of the methodology of this analysis to significantly improve clarity and detail (L451-527). As part of this, we have included a new explanatory figure (Figure 4) to help communicate the steps of the analysis to readers.

5. I was surprised not to see any discussion of sampling. I believe interactions are easier to miss than species when field sampling – is this true? I would appreciate the author's perspective on how to conserve something (ie interactions) that can be difficult to measure? Through another lens pertaining to this study, how confident are authors that they have captured interactions in their communities and that vulnerability is not influenced by sampling completeness? It is common for network studies to show rarefaction plots and I think this study may benefit and hopefully be stronger by presenting information on the sampling completeness of their data sets.

13. Response: We agree with the Reviewer that sampling is an important issue that warrants discussion in this paper. There is some evidence that interactions are easier to miss than species when sampling. For example, Chacoff et al (2012, Journal of Animal Ecology, 81, 190-200) found that "In spite of our high sampling effort, and although we sampled 80% of the pollinator fauna, we recorded only 55% of the interactions. Furthermore, although a 64% increase in sampling effort would suffice to detect 90% of the pollinator species, a fivefold increase in sampling effort would be necessary to detect 90% of the interactions." Although there is also evidence that much higher proportions of interactions can be sampled (e.g. ~ 70%, Traveset et al., 2015, Nature Communications, 6, 6376), and rarely more than 50% is missed. While much of the advice on how to conserve interactions that are difficult to detect is very much the same as for species, for which there is a large literature of field sampling methods and statistical techniques e.g. rarefaction, there have recently been some developments using Bayesian hierarchical models to infer missing

interactions in network data (e.g. Graham, and Weinstein, (2018), Ecol Lett, 21: 1299-1310). These approaches are likely to be very useful for interaction conservation and overcoming this issue with missing interactions during sampling. Often these approaches require networks as time series, so it is important that field researchers retain these data for their analyses, rather than aggregating all time slices into a single network. We have added discussion of these issues in L311-319.

We tested the Reviewer's idea that vulnerability may be influenced by sampling, but we did not find a significant relationship between log of a network's sampling intensity (calculated following Schleuning, M. et al. 2012. Curr. Biol. 22: 1925–1931, and Dalsgaard et al., 2017, Ecography, 1395-1401) and its median vulnerability ($P = 0.0503$). While this is approaching significance, the R^2 was low (R^2 = 0.07) and the relationship was driven by a handful of outlier points (identified using Cook's distance with a threshold of 4/n, where n is the sample size). When these were removed, the relationship was even less significant ($P = 0.115$, R^2 = 0.04). We have added this analysis to the supplementary information (Analysis S1) and referenced it in the main text (L318-319).

Following the Reviewer's suggestion, we have added information on the sampling intensity of our networks (L332-333).

Specific comments:
6. I recommend a small tweak to the first paragraph. To me, ecosystem function does not happen without biotic-abiotic interactions/feedbacks yet the current text focuses uniquely on the biotic component. I recommend the author consider either infusing the abiotic component in here or dropping the ecosystem language.

14. Response: We thank the Reviewer for raising this point - we have added mention of biotic-abiotic interactions to the first paragraph (L41-43).

7. Lines 59-66. This is a really nice justification for the focal data.

15. Response: We thank the reviewer for this positive comment.

8. Lines 70-71. The conservation planning literature has key concepts of vulnerability and irreplaceability. (see Margules & Pressey 2000 Nature). I see parallels here. Given the interest in informing conservation, I wonder if the authors should explicitly explore the parallels between their ecological measures of vulnerability and importance and conservation metrics of vulnerability and irreplaceability?

16. Response: We agree with the Reviewer that there are definitely parallels between our measures and the measures of vulnerability and irreplaceability from the systematic conservation planning literature. We are pleased the Reviewer noticed this parallel as it was one of the original ideas that inspired this work.

In the conservation planning literature, irreplaceability is usually defined in the context of reserve selection algorithms and thus an explicit comparative analysis is not possible here.

Additionally, we feel a full analysis is beyond the scope of the work and would dilute our central thesis. However, we agree that this point is definitely worth including and so we have added text that highlights the parallels between these two sets of metrics in L83-86.

9. Fig. 2. P-values are not particularly informative when we have such large sample sizes. I recommend the authors report more meaningful measures of effect size like correlations or R^2. I suspect these metrics will be fairly small despite being "statistically significant".

17. Response: We have added R^2 values for the correlation in Figure 2 (L148). We calculated both the marginal $R^2_{GLMM(m)}$, which represents the variance explained by fixed effects, and the conditional $R^2_{GLMM(c)}$, which represents the variance explained by both fixed and random effects ($R^2_{GLMM(m)} = 0.20$, $R^2_{GLMM(c)} = 0.42$, Nakagawa and Schielzeth 2013, Emer et al. 2016, Kaiser-Bunbury et al. 2017, Bartoń 2018 - see bibliography in manuscript).

Reviewer #3:

The authors study the relationship between link vulnerability and its contribution to network feasibility in a set of empirical networks. Then, they use information on species' taxonomic classification to test whether or not these two link metrics are evolutionary conserved. The paper is well written and the analyses are well conducted. In addition, I do believe that this is novel and publishable research. However, albeit useful to study network structure, I believe that feasibility might not be the most appropriate way to understand the "importance" of ecological interactions. Moreover, I also fear that the terminology used by the authors might overplay the actual novelty and importance of the results. The major concerns that I had regarding the paper are listed below:

18. Response: We thank the reviewer for their comments on our manuscript and address the concerns where they are raised specifically below. Specifically, in the responses below we address the issue of importance and the terminological concerns the reviewer mentions.

First and foremost, the manuscript left me wondering how surprising the positive relationship between link "vulnerability" and "importance" actually is. For example, I would like to see the relationship between "importance" and standardised generalisation. I would expect the removal of links from low degree species to condition more strongly feasibility than the removal of links from highly connected species. This is because by removing links from poorly connected species you are limiting the resources of resource-limited species, which strongly conditions their existence on their few remaining links. In other words, I would expect species with high degree being more robust to the removal of one of their links than species with low degree. In this context, I would then want to know if the species lost following the removal of highly vulnerable links are those same species that have had their links removed. I understand that this would require simulations, which the authors
avoid by using the approach by Saavedra el. al. 2016. However, I find the result "highly connected species are more robust to link removal than poorly connected ones" much less surprising than "vulnerable links are the most important ones". Along similar lines, the link contribution to feasibility seems to me a bit superficial for assessing the actual "importance" of an ecological interaction. I would argue that the identity and number of species lost

following link removals is information that is clearly needed for characterising the "importance" of a link.

19. Response: The Reviewer discusses link removal in the context of estimating persistence: where a perturbation is introduced and a simulation is run to see how many (and which) species go extinct. As we responded to a similar concern raised by Reviewer 1 (response #3), we agree that highly-connected species being more robust to link removal than poorly connected ones might not be a surprising result when measuring persistence. However, as mentioned by the Reviewer, here we do not consider whether species are lost following link removal; instead, we consider feasibility, which is the range of conditions under which all species in the network can coexist. Thus, if two species lose their link, the question is not whether those species will be lost, but whether the loss of this link results in a larger or smaller range of conditions under which all species in the network can survive. Thus, (as we noted in #3) removing a link of a species could lead to the extinction of that species in a persistence framework, but the overall effect on feasibility in the remaining community may increase or decrease and this is not so obvious to deduce. Indeed, no explicit relationship between persistence and feasibility is to be expected (pers comm R Rohr).

This complexity is evident when looking at the correlation between standardised generalisation and importance (which we now include in Figure S2 as recommended by both Reviewers 1 and 3). The overall trend is that specialised links are important and generalised links are not important, but there is a substantial amount of noise in the relationship: there are specialised links which are not important, and highly generalised links that are important (Figure S2). There are even cases in our results where removing a specialised link *increases* the feasibility of the network (Figure S2), which is the opposite to what would be expected by the Reviewer (if specialist links are important, their removal would be expected to decrease feasibility). These cases highlight the difficulty of assuming an immediately-intuitive relationship between link generalisation (or vulnerability) and importance where specialist links (or low vulnerability links) are important and generalist (or vulnerable) links are not important. Indeed, our model still leaves 40% of variance in importance unexplained by either vulnerability or the network identity random effect. There is clearly further research needed to fully understand the relationship between these properties and to characterise the noise in our data. However, we do think the Reviewer's comment that "by removing links from poorly connected species you are limiting the resources of resource-limited species, which strongly conditions their existence on their few remaining links" could be useful for understanding the relationship between vulnerability and importance and so have added it to the discussion (L237-240).

We choose to focus on the feasibility measure, rather than the estimates of persistence that the Reviewer mentions, because the feasibility measure saves us from a fundamental challenge. Estimating persistence requires as starting point a feasible community. This means that we need to find a parameterisation where all species are present (feasible = nonzero positive abundances) before we perform a perturbation to estimate after simulation how many and which species persisted. The larger the community, the harder it is to find conditions of feasibility, and even when found there are likely to be many possible parameter settings to choose as starting points for our persistence simulations. The problem is that

results are very sensitive to the choice of parameters - by changing the intrinsic growth rates, it is possible for almost any network structure to have full persistence (Rohr et al 2014, Science). Thus if the model is parameterised arbitrarily without empirical data, studies can find inconclusive results that cannot be generalised, because the parameterisation drives much of the results. So which parameter setting should be chosen? One solution is to choose as many sets as possible and repeat the analysis to get average persistence estimates. This is obviously a time consuming and still incomplete approach (the parameter space can be too large to sample adequately), especially when one wants to compare persistence across multiple networks.

There is also an additional problem in the form of how to specify the perturbation. Our question is about how the removal of a link affects the ability of species to survive in the presence of a perturbation. Thus an analogous version of our analysis in a persistence framework would be to measure persistence in networks with and without a focal link. In other words, for networks with and without a focal link, run persistence simulations with a particular perturbation (note that the link removal itself is not the perturbation) and compare the proportion of species that survived. However, problematically, there is now strong evidence that network persistence is highly dependent on the type, direction and magnitude of the perturbation specified. A paper by Cenci et al (2017, Ecology and evolution, 8(14), pp.6852-6859, doi: 10.1002/ece3.4252) investigated this issue by generating networks with either modular or nested structures, and then measuring species persistence under a variety of perturbations. They found that, depending on the type and direction of the perturbation, either modular structures appeared more persistent, nested structures appeared more persistent, or both structures appeared equally persistent. In other words, because the results of a persistence simulation can depend so strongly on the perturbation specified, it is very difficult to conduct a systematic analysis that is comparable between networks and which generates generalisable results about the relationship between network structure and dynamics.

Instead, the feasibility framework we follow avoids the problem of choosing a single set of parameters (e.g. intrinsic growth rates) by considering the size of the domain of intrinsic growth rates that leads to feasibility. It also avoids the issue of having to specify a particular perturbation: instead of knowing how species respond to stress in a particular direction, we instead study the volume of the space where species coexist when stress is moved in any direction. In other words, the question becomes not whether we can find a feasible equilibrium point for a fixed set of parameter values, but instead asking how large is the range of parameter values that are compatible with the stable coexistence of all species. In this way we use a standardised and comparable protocol from which we can generalise about the impact of network structure. Nonetheless, we agree with the Reviewer that our motivation for using the feasibility approach was not clear, and readers may wonder why we did not use other ways to measure importance. We have added a discussion explaining our choice of feasibility as the response measure (L138-145; L369-396) and we have also clarified the term ‘importance’ in the manuscript and define it explicitly as feasibility contribution throughout the manuscript (L26-27, L133, L159-160, L198-199, L426-427). We believe this emphasises how ours is just one measure of link importance, but there may be other properties of community stability that could be used. In line with this we have also

added a discussion to the main text, where we state that feasibility is just one aspect of importance, and that other definitions could be used, pointing readers to two papers that catalogue the wide range of stability measures available, including those based on the identity and number of lost species in simulations i.e. persistence (L138-140). We have also added a similar section to the discussion (L292-295).

Regarding the second part of the manuscript, I would also want to break down the results to try to understand them better. For example, I would like to see if standardised generalisation is also "evolutionarily conserved". If so, it would basically mean that the number of interactions that species have is evolutionarily informed. I would expect this to be true given existing work on the phylogenetic signal underlying species interactions in mutualistic networks (as just one example different from the ones given in the manuscript, see Rezende et. al. Oikos 2007). Complementarily, I would also like to see if standardised frequency is "evolutionarily conserved", which I would also expect to be true (but I would find more interesting as I don't recall any other work on this).

20. Response: We agree that it is useful to include results for the consistency analysis for standardised frequency and generalisation. We have now run this analysis and found similar patterns of consistency in frequency and generalisation as for vulnerability and importance, where frequency and generalisation tend to be conserved. We have included the results in the Supplementary information (Figures S4 and S5), and referenced them in the main text (L173, L254, L524). We have also included the recommended reference by Rezende et al (2007) in the main text (L258).

I am also troubled by the terminology chosen by the authors. In particular, I find "importance" to be a misleading term, as it is very subjective and might overplay the authors' results. Imagine that we have a feasible network for a range of conditions A, and that removing a vulnerable link leads to feasibility conditions B, where B<A. Imagine then that the difference in the feasibility conditions basically leads to the loss of one species (e.g. one of the species involved in the removed link). Now, let's assume that removing a link with low vulnerability leads to feasibility conditions C, where B<C<A. In this case, however, the difference in the feasibility conditions leads to the loss of 10 species. Despite B<C, one could argue that the removal of the link with low vulnerability is much more important as it leads to the loss of many species. In other words, the term "importance" is not really accurate (and unnecessary), as the authors could just basically use feasibility
contribution (as defined in line 313).

21. Response: Feasibility is the range of conditions under which *all* species survive in a network. Thus, using the Reviewer's example, all species would survive under feasibility conditions A, B and C. This means that removing a link with low vulnerability reduces the size of the feasibility domain but it cannot lead to 10 species being lost (as in the example of C above). Instead, C would simply be the range of conditions under which all species survive in the network following the removal of the low vulnerability link. All species survive within all feasibility domains, the question is whether the range of conditions compatible with species coexistence is smaller or larger after removing a link. We have now clarified this point in the text (L393-396).

However, the Reviewer is right that the feasibility domain only specifies the conditions where all species coexist. Thus if a community leaves the feasibility domain, this will result in extinctions, but we do not know how many. This is because of the way feasibility works – if a perturbation pushes a community out of the feasible domain, this could lead to 5 species being lost. However, a different perturbation could lead to 1 species being lost. Conclusions about species persistence using simulation approaches are highly dependent on the type and direction of the perturbation (see response #19 above and Cenci et al (2017, Ecology and evolution, 8(14), pp.6852-6859, doi: 10.1002/ece3.4252). Feasibility avoids this issue by studying the size of the feasibility domain - instead of knowing how species respond to stress in a particular direction, we instead study the volume of the parameter space where species coexist when stress is moved in any direction, making feasibility a much more general measure and one we prefer for this study (see response #19 for a more extensive discussion of these issues).

As we discussed in response #19 we choose to use feasibility as the measure of importance as it is a much more general measure than species persistence. Thus we choose to keep the term 'importance', as we believe as it is a clearer and more concise way of expressing the feasibility measure, that captures its meaning for our analysis rather than simply being a technical term, and it is a term that directly ties into the 'risk' concept we introduce in the introduction. However, we agree that it is important to clarify that this is not the only way of measuring the importance of a link and that our definition is subjective and definitely not an all-encompassing measure of importance. Therefore we have added a discussion where we state that feasibility is just one aspect of importance, and that other definitions could be used, directing readers to two papers that catalogue the wide range of stability measures available, including those based on the number of lost species in simulations i.e. persistence (L138-140). We have added a similar section to the discussion (L292-295). We have also explicitly defined importance of feasibility contribution throughout the manuscript (L26-27, L133, L159-160, L198-199, L426-427) to clarify that ours is just one definition of importance and to remind readers of the technical and specific definition of the term in our manuscript. We have also substantially expanded the explanation why we choose feasibility as the response measure (L138-145; L369-396) so that readers can understand the motivation for choosing feasibility over other approaches e.g. persistence.

Finally, the network representation in Figure 4 is totally unnecessary. Why is a depiction of a network useful here? If the information that the authors care about is the percentage of links of each type (i.e. links that are "highly vulnerable and highly important", links that are "not vulnerable and not important",...), visualising this information in a network form is not useful (it very rarely is). In other words, four numbers can convey the same amount of information (potentially more, as Figure 4 only depicts the results for one network).

22. Response: We agree with the Reviewer and have removed the figure and references to it.

Minor comments

- I found the distinction between "interaction" and "link" a bit confusing in line 81. It might be worth giving examples.

23. Response: We agree with the Reviewer that our definition of these terms was unclear. We have now reworded the definition to include the ‘metaweb’ and ‘local’ web terminology suggested by Reviewer 2, and have added a much clearer example to explain the terms (L71-78).

- How do you reconcile the observed variability of plant-pollinator interactions (Caradonna 2017 Ecology Letters) and the results you found?

24. Response: As shown by Caradonna (2017) and others, plant-pollinator networks have significantly topological variation over time, on daily, weekly, monthly and yearly timescales. This means that specific species-species links form and dissolve over time as species rewire their connections.

Despite this continuous rewiring, a number of studies have found evidence that whole-network structure can remain stable over time (e.g. Petanidou et al, 2008, Ecology Letters; Dáttilo et al, 2013, Oikos). Similarly, evidence from antagonistic networks suggests that species' roles in networks can be consistent over space and time, even in the presence of species turnover and rewiring (Baker et al., 2015, Ecography).

We find similar results to those in Baker et al’s (2015, Ecography) study, but considering link roles (in terms of vulnerability and importance) rather than species roles. We find that the roles of links that occur across multiple communities can be consistent, despite spatial turnover in the identities of the other interactions and species present. Thus while rewiring means that links can form and dissolve over time, our results suggest that when the links are formed, they tend to have similar properties, in terms of their vulnerability and importance. This implies that, despite network structure changing over time and space, link consistency imposes multiple constraints on network structure because the roles of links tend to be consistent.

We agree with the Reviewer that this is a useful issue to raise and have added a discussion of this issue to the main text (L267-274).

- Fig. 2 is missing the confidence intervals for the regression.

25. Response: We have added confidence intervals.

- Wrong notation in line 328

26. Response: We have fixed the notation in this line (swapping ‘a’ for alpha) (L407 in the revised manuscript).

- Stability is not the same as feasibility. The authors need to be consistent (as just one example, see line 175).

27. Response: We thank the Reviewer for mentioning this, as we agree it could cause confusion. We were referring to feasibility as structural stability (as opposed to dynamical stability), but agree this is confusing, and so we have changed the text to now use the term 'feasibility' throughout the main text and supplementary information.

- The caption for figure 2 and 3 should clearly state that this is the absence of interspecific competition.

28. Response: We have added that the results are in the absence of interspecific competition to the figure captions (L162-164, L205-207).

- Along similar lines, why do the authors need to use the mean field approximation for the intraguild competition parameters? I wonder if the results hold without this assumption.

29. Response: In the absence of empirical information about interspecific competition, such an approximation is a logical and standard approach - it is the simplest method with the fewest assumptions. This is also the framework recommended by Rohr et al (2014, Science, 345(6195), p.1253497) where the feasibility measure for mutualistic systems was introduced. We note that the results in the main text do not include interspecific competition (L415-416), so the mean field approximation is not used for these results - it is only used for the simulations which include interspecific competition in the supplementary material. However, we agree it is important to discuss these issues, and so we have added an explanation of this topic in the main text (L405-409).

Reviewer #4:
[identifies himself as Ignasi Bartomeus]

I really enjoyed reading this manuscript, as is it presents clear results, is extremely well written, and is intellectually stimulating. The manuscript shows that two network metrics depicting interaction vulnerability and interaction importance are correlated and suggests conservation implications of this finding. The analysis in itself is robust and uses state of the art methods, however, given the complexity of measuring things such as vulnerability and importance from static representations of interaction networks, most of my comments are centred on how can we interpret this results and trying to understand potential confounding factors that can influence the findings.

30. Response: We thank the Reviewer for his positive comments about the manuscript and address his concerns below.

I also miss a clear statement on why focusing on link loss, and what this means. As I understand, a link can be lost if one of the partners are lost, but I don't think this is what you are interested in. A link can also be loss if two species stop interacting for whatever reason (e.g. phenological decoupling, changing behaviours, …). I think this is the scope of inference of your paper, and should be clear stated early on. Correct me if I am wrong.

31. Response: The Reviewer is right that this is our focus. We have now clarified this in the introduction (L65-70).

First I will comment about the measures used.

Vulnerability:
I would like to know the authors opinion on how to take into account interaction asymmetries. If I understand correctly, your measure of generalisation treats equally a symmetric pair of species interacting (3 links each) than an asymmetric one (5 links one species, 1 link the second one). In my opinion, the second scenario shows a less generalised link.

32. Response: We agree with the Reviewer that interaction asymmetry is an important consideration. However, when exploring the properties of asymmetry (i.e. the ratio of the degree of species 1 to the degree of species 2), we found that, for a given species, the asymmetry and generalisation of its links are perfectly collinear (or have a curved relationship depending on which way the ratio is defined). Specifically, the slope of the relationship between the generalisation of the links of a species and the asymmetry of the links of a species is always equal to half the degree of the species.

Upon exploring further, we were able to derive a proof for this mathematically:

First, some terms
g = generalisation (g_{uv} = generalisation of link between nodes u and v)
a= asymmetry (a_{uv} = asymmetry of link between nodes u and v)
deg = degree

Let's pick a node, u, in the network, keep it fixed for the analysis (for the example, this can be the species represented by the first column in the bipartite matrix).
For any neighbour v of u (i.e. any node connected to u; a 'partner' in ecology terms), we have

$g_{uv} = (deg(u) + deg(v)) / 2$

$a_{uv} = deg(v)/deg(u)$ (v is the row and u is the column of the bipartite matrix)

So what does it mean that they are collinear?
It means $g_{uv} = \lambda \cdot a_{uv} + c$ (i.e. $y=mx + c$, this is what we want to show: that g is a linear function of a)

Here is the important bit: $\lambda = \lambda_u$, $c = c_u$
So λ and c can depend on u (our column), but not on v - since the slope and intercept need to be the same for any neighbour of u. (But you'll get a different line if you do the analysis for a different column, i.e. a different u).

Let's say we have another neighbour of u, call it w. We again want to have
$g_{uw} = \lambda \cdot a_{uw} + c$

Where

$g\_uw = (deg(u) + deg(w)) / 2$

$a\_wv = deg(w)/deg(u)$

g_uw and a_uw can be different from g_uv and a_uv but λ and c need to still be the same

Remember the slope of a line is calculated as $\Delta x/\Delta y$

Let's calculate this here (g is our y, a our x)
$\lambda = \Delta g/\Delta a = (g\_uw - g\_uv) / (a\_uw - a\_uv) = 0.5 * (deg(w) - deg(v)) / ((deg(w) - deg(u)/deg(u)))$
$= 0.5\ deg\ (u)$

Note this step would not work so nicely if the quotient in a was reversed (hence a curved relationship between g and a if the ratio is inverted).

So the gradient is determined solely by the degree of node u (the column node) and is independent of v and w (the row nodes)!

We've shown:

$g\_uv = 0.5deg(u) \cdot a\_uv + c$
For *any* neighbour ('partner' in ecology terms) of v and u (i.e. also for w).

We're now done, but just for completeness/fun we can compute c as well.

$c = g\_uv - 0.5 * deg(u) * a\_uv$

$= 0.5 * (deg(u) + deg(v)) - 0.5 * deg\ (u) * deg(v)/deg(u)$

$= 0.5\ deg\ (u)$

So in this case $c = \lambda$

Going back to our original formula: $g\_uv = \lambda \cdot a\_uv + c$

We now know: $g\_uv = 0.5deg(u) \cdot a\_uv + 0.5\ deg(u)$

This can actually be proven by simple algebra:

$0.5deg(u) \cdot a\_uv + 0.5\ deg(u)$

$= 0.5\ deg(u) \cdot deg(v)/deg(u) + 0.5deg(u) = 0.5[deg(u) + deg(v)]$

$= g_{uv}$

Thus due to the intrinsic mathematical collinearity between link generalisation and link asymmetry, we have not included it in our analysis. But we thank the reviewer for raising this point as we did not realise this would be the case initially and this was fun to think about. We have also added a note in the main text stating that asymmetry and generalisation are collinear (L154-157).

In a similar though, I understand pretty well species generalisation, but applying that concept to links is harder. For example, while more generalised species should be less vulnerable, more generalised links may simply be “redundant”.

33. Response: We agree with the reviewer that referring to the links as generalised can initially be hard, given the well-established use of the term ‘generalisation’ for species. However, while we agree ‘redundant’ could be a suitable alternative term, we prefer to retain the original term of ‘generalisation’ in order to be consistent with the name used in the original definition of the metric by Aizen et al (Science, 2011, Vol. 335, Issue 6075, pp. 1486-1489). We feel that it is useful to keep the term consistent through the literature in order to avoid confusion with multiple terms referring to the same concept, especially as link generalisation is a relatively new concept. We also like the term generalisation because of its relationship with species generalisation – link generalisation is a derived property of species generalisation and so we feel this works well and will be familiar to readers and that changing it could cause confusion. However, following the Reviewer’s suggestion, we now mention the term ‘redundant’ to aid understanding when we introduce the generalisation metric (L130).

Importance:
I like the use of novel structural stability measures, but I would be cautious on its interpretation, as the feasibility domain calculated is just part of the story. I agree that it is relevant, but larger feasibility domains may exclude some species depending on its r values, and smaller feasibility domains may allow for the coexistence of the full community. I am not asking for any specific change here, but maybe clarifying this in methods would help.

34. Response: We think that the Reviewer may have misunderstood the feasibility measure here. The size of the feasibility domain is the volume of the parameter space where all species survive. Thus while a small feasibility domain means there is a small range of conditions (r values) under which all species survive, and a large feasibility domain means there is a large range of conditions under which all species survive, there is always complete coexistence of all species within the feasibility domain regardless of its size. We have clarified this in the methods to ensure it is clear to readers (L393-396).

Second, I feel that the authors make too much emphasis on informing conservation measures for models showing a low predictive power, and not validated empirically (e.g. line 58). For example, the use of structural stability relies on many assumptions (e.g. mean field

competition, static interaction networks), and while it may be a good theoretical proxy, I would be cautious when using it to inform conservation. Furthermore, we know a bit on how to protect species, but is unclear to me how we could protect links e.g. Line 250.

35. Response: We agree with the Reviewer that we should reduce emphasis on informing conservation. We have done this on line 58 from the original manuscript as suggested (L57 in the revised manuscript - reference to conservation has been removed) and throughout the text, either by deleting phrases or using more cautious language, such as a 'may', 'starting point', 'could' or 'in the future' (e.g. L32-33, L275). We have also added a substantial amount of text to the discussion in the paragraph on conservation implications. This new text states that it is too early to use these methods for conservation until empirical validation occurs and assumptions are tested, and that currently our results provide a good theoretical proxy that must be tested further (L290-305).

With respect to the Reviewer's comment about how we should protect links: we had a discussion on link conservation compared to species conservation in the original manuscript (L306-319 in the revised manuscript). In response to reviewer's 2 comment (response #13), this has now been expanded to include the issue of sampling, which will also play an important role in link conservation on the ground (L306-319). We agree that including this discussion at the end of the manuscript might make it easy to miss as simply a concluding paragraph. We have therefore separated the paragraph from the conclusion (L306-319), and referenced it at the relevant point in the text (L250 in the original manuscript, L278 in the revised manuscript) that the Reviewer mentions.

Finally, I would suggest exploring how species abundance may be driving the results. The results are convincing, but given the complexity of the metrics used it would be good to discard confounding factors that may be driving both metrics.

36. Response: The reviewer touches upon a sensitive and recurring point that troubles the analyses of network patterns and relationships. Because we do not have independent abundance data for species in our networks, we cannot explicitly test the influence of abundance. The best we can do is to use total interaction frequency as a proxy for abundance, but this is not always a good proxy (e.g. Vizentin-Bugoni et al., Proc. R. Soc. B 2014 281, 20132397), and particularly for our analysis there is unavoidable circularity because the vulnerability metric uses interaction frequency in its calculation, though the importance metric is independent of interaction frequency.

However, we agree it is important to at least try to assess how species abundance could affect results, even if total interaction frequency is not necessarily a good proxy for abundance.

To do this, we used a null model approach. In this approach, many null networks are generated and compared to the empirical network to make inferences about the factors that govern the empirical network's structure. While this approach is powerful, it is also computationally costly, as analyses have to be repeated for each of the many null networks.

This is problematic for our project, which already has high computational demands: 41 networks with an average of 106 links, with importance values calculated 1000 times for each link (due to stochasticity in its calculation), for both competition and no competition requires 8,692,000 simulations (41*106*1000*2), which took a substantial amount of time to run even when heavily parallelised on a high performance cluster. Repeating this analysis for 1000 null versions of each of the 41 networks increases the number of simulations required to 8,692,000,000, which is infeasible. Thus, instead, we carried out an exploratory analysis on one representative network, close to the median size and connectance of networks in our data: size = 52, connectance = 0.15 (dataset median size = 47, dataset median connectance = 0.16). While not ideal, we believe this analysis can at least help identify whether abundance influences our results.

For our representative network, we generated 100 null networks, using a null model that assumed interaction neutrality by assigning interactions according to a probability matrix, A, where element a_ij was the relative abundance of animal species i multiplied by the relative abundance of plant species j (Vázquez et al. 2007, Maruyama et al. 2014, Vizentin-Bugoni et al. 2014, 2016). Therefore, the model assumes that two species with high abundance have a greater likelihood of interacting than two species with low abundance. The model constrained the number of links to be the same as that in the empirical network and ensured that each species had at least one interaction (Vázquez et al. 2007). We used total interaction frequency as a proxy for abundance. This model therefore represents how the network would look if abundance was the only factor governing interactions. If the model can accurately reproduce our results, then we can conclude that our results were driven by abundance. If it does not accurately reproduce our results, then we can conclude that our results were not driven by abundance and other factors must play an important role.

For each of the 100 null networks, we calculated: (i) the mean link vulnerability, (ii) the mean link importance, (iii) the standard deviation of the link vulnerability, (iv), the standard deviation of the link importance, (v) the slope of the relationship between vulnerability and importance. We compared these values to the equivalent values from the empirical version of the network.

Our results were as follows:

Mean link vulnerability
95% CI of null network values: 0.47 – 0.55
Empirical value: 0.45

Mean link importance
95% CI of null network values: -1.40 – -0.54
Empirical value: -0.003

Standard deviation of the link vulnerability
95% CI of null network values: 0.27 – 0.32
Empirical value: 0.24

Standard deviation of the link importance
95% CI of null network values: 0.067 – 0.093
Empirical value: 0.049

Slope of the relationship between vulnerability and importance
95% CI of null network values: -0.089 – -0.01
Empirical value: 0.08

We found that for none of the metrics did the empirical value overlap the 95% CI of values generated by the null model. Thus from these results of a representative network it does not appear that abundance drove the vulnerability or importance values, or the relationship between vulnerability and importance. We report on the importance of abundance as explanatory variable to our patterns (also from point #39 below) in our discussion (L251-254) and include the above results in the Supplementary information, Analysis S2.

In line comments:
- line 124: Please, give also estimates and SE.

37. Response: We have added these (L147).

- line 147-151: I took me a few reads getting that the first % refer to all positive values, and the second ones to only the statistical significant ones. Maybe it was me, but you may clarify the writing to ensure no-one else gets lost here.

38. Response: We have reworded this section to improve clarity (L183-191).

- line 154-155: As alternative explanation is that abundant species (those with more and more frequent links) tend to be abundant elsewhere?

39. Response: We agree that this could be an explanation. While we cannot test this explicitly (response #36), as we do not have data on species abundances for our networks, we have revised the discussion to emphasise this point (L251-253). Additionally, following a comment from another Reviewer (response #20), we now report the results of the consistency analysis for frequency (a possible proxy for abundance) in the supplementary material (Figures S4 and S5) and reference this in the revised main text discussion (L253-254). We find similar patterns of conservatism in frequency as vulnerability and importance, suggesting that consistency in abundance could help explain the consistency in vulnerability. However, we note that abundant species do not always have more frequent links (e.g. Vizentin-Bugoni et al., Proc. R. Soc. B 2014 281, 20132397), so there could also be other processes at work (such as consistency in generalisation, which we also find evidence for - Figures S4 and S5). We also note that this abundance/frequency explanation is only relevant to the consistency of vulnerability (which is composed of measures related to frequency and generalisation) but not importance, as importance values are governed by the whole network structure, not just the roles of the two partner species involved in the link. Thus, the result that link importance is consistent across occurrences suggests that links and their partner species are embedded in networks in similar ways even in different contexts. In

other words, link consistency imposes multiple constraints on the roles observed within a network and, consequently, community structure.

- Paragraph starting at 168 is now quite redundant with Figure 4. Maybe you can explain how representative is this particular network. For example, the proportion of links per category is similar across all networks?

40. Response: We have removed Figure 4 and references to it (including the paragraph referenced by the Reviewer) in response to a comment from Reviewer 3.

- line 215: I am confused, because you use “only simple topological information” to calculate structural stability, right? Even if the models are complex, the baseline information is the same.

41. Response: We agree the baseline information is the same and so have removed this sentence.

Line 273: Data section: I miss here a description of how links are measured. Total visits? Visits per flower? A combination of both? And how this matters for the analysis.

42. Response: We have added a description stating that for the majority of networks (we could not access a small number of original data sources as these were in e.g. theses), link weights were equal to total visits (L328-330). To control for any variation in study design or methodology, we include network identity as a random factor in our analysis, so this is accounted for in our model.

Eq 321: Basically, I understand that you use the topology to create the mutualistic effect, and assume a mean field competition among species, but is unclear to me how do you add the intrinsic growth rates. Where are those coming from?

43. Response: The strength of the feasibility approach is that it does not rely on a single set of intrinsic growth rates. This eliminates the need for difficult (and often arbitrary) parameterisations of these variables (see also #19).

Instead, the dynamics model is solved to acquire all possible combinations of intrinsic growth rates that lead to fixed positive abundances of all species (i.e. that satisfy the feasibility criterion). Full details are in e.g. Rohr et al (2014, Science, 345(6195), p.1253497. Thus the feasibility approach considers all combinations of growth rates, not just one particular set of values.

We agree it’s important for this part of the method to be explained to readers, so we have added an overview of this explanation to the main text (L419-426).

Also in 324 change the notation of one of the two “gamma_ij” in gamma_ij = gamma0*gamma_ij/… as I understand that gamma_ij is used in two different contexts.

44. Response: We have changed the second gamma_ij to L_ij to improve clarity (L403)

Best,
Ignasi Bartomeus
