# Supplementary material for: Estimating the risk of species interaction loss in mutualistic communities
Source: PLoS Biol. 2020 Aug 31;18(8):e3000843. doi: 10.1371/journal.pbio.3000843 (PMC7485972; doi:10.1371/journal.pbio.3000843)
Supplement: Attachment — Submitted filename: review_simmons.pdf [file pbio.3000843.s014.pdf]

In mutualistic networks, are links between generalists more or less important than links connecting specialists?

May 6, 2020

Abstract

These are technical notes concerning a review of a manuscript by Simmon's et al for PLOS Biology. In the first section I propose a calculation of the mutualistic benefit of a given link. In the second section I justify the ecological relevance of the first section using a Lotka-Voltera model. In the third I discuss the notion of generalism of a link and how this could relate (or not) to its vulnerability. I then conclude that vulnerable links can be the most important ones, but only is particular condition identified in this document.

1 Mutualistic benefits around a focal link

Consider a network of mutualistic interactions, denoting A_{ij} the (positive) directed interaction from node j to node i. We denote by d_j the degree of node j. We are interested in the effect, on the whole network, of removing (or simply decreasing) the interactions between two nodes k and l (red link bellow).

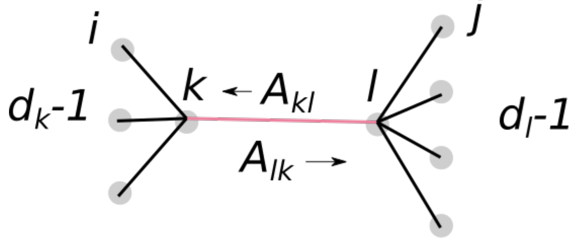

In the figure we do not show links going from nodes i and j to other nodes, but we do assume they exist. Why we focus on the above motif will be justified in the next section. Consider the effect $\Delta_{\leftarrow}$ of removing A_{kl} on overall mutualistic strength in the above motif. We need to sum various contributions. First there is the effect directly felt on node k from l, proportional to A_{kl}. Then there is the sum of all nodes j via node l on k, proportional to $A_{kl}A_{lj}$ ($d_l - 1$ source nodes).

There is the effect on node i from l, proportional to ($d_l - 1$ target nodes). Then there are effects from nodes j to i via l and k, proportional to $A_{ik}A_{kl}A_{lj}$ ($d_l - 1$ sources and $d_k - 1$ targets). If we sum all these effects we get

$$\Delta_{\leftarrow} = A_{kl} + \sum_{i=1}^{d_k-1} A_{ik}A_{kl} + \sum_{j=1}^{d_l-1} A_{kl}A_{lj} + \sum_{i=1}^{d_k-1}\sum_{j=1}^{d_l-1} A_{ik}A_{kl}A_{lj}$$

$$\Delta_{\leftarrow} \approx A_{kl} + d_k \langle A_{ik}\rangle A_{kl} + d_l \langle A_{lj}\rangle A_{kl} + d_k d_l \langle A_{il}A_{lj}\rangle A_{ik}$$

where we approximated $d_{k,l} - 1 \approx d_{k,l}$ and wrote $\langle A_{ik}\rangle$ for the average strength from k to i, $\langle A_{lj}\rangle$ the average strength towards l and $\langle A_{il}A_{lj}\rangle A_{ik}$ the average strength from j to i via k and l. We now suppose that interactions are weaker when coming from a node with many links. Concretely, we set

$$A_{ij} = \gamma d_j^{-\delta};\ \delta \geq 0;\ \gamma < 1$$

Then the above expression for total change in mutualistic benefit reads:

$$\Delta_{\leftarrow} \approx \gamma d_l^{-\delta} + \gamma^2 d_k^{1-\delta} d_l^{-\delta} + \gamma^2 d_l^{1-\delta}\left\langle d_j^{-\delta}\right\rangle + \gamma^3 (d_k d_l)^{1-\delta}\left\langle d_j^{-\delta}\right\rangle$$

We can make the same reasoning for the interaction going right, from k to l and by summing the two we have the total reduction $\Delta = \Delta_{\leftarrow} + \Delta_{\rightarrow}$ of mutualistic strength in the represented motif, if $C_\delta = \left\langle d_j^{-\delta}\right\rangle$ (approximated as a constant in what follows)

$$\Delta \approx \gamma\left(d_l^{-\delta} + d_k^{-\delta}\right) + \gamma^2\left(\frac{d_k + d_l}{d_k^\delta d_l^\delta} + C_\delta (d_l^{1-\delta} + d_k^{1-\delta})\right) + 2\gamma^3 C_\delta (d_k d_l)^{1-\delta}$$

The question is: which are the links that contribute the most in terms of mutualistic benefits? Without the trade-off, i.e. if $\delta = 0$ (all links have the same strength) then loosing the link between node k and l leads to the loss

$$\Delta \approx \gamma + 2\gamma^2 (d_l + d_k) + 2\gamma^3 d_k d_l$$

and we see that the larger d_k and d_l the bigger the loss. **Thus links between the most connected nodes are the most important.** On the other hand, if $\delta = 1$ (strong trade-off) then

$$\Delta \approx \gamma\left(d_l^{-1} + d_k^{-1}\right)(1 + \gamma) + 2\gamma^2 C_1 + 2\gamma^3 C_1$$

the larger $d_l^{-1} + d_k^{-1}$ (which behaves as an inverse average degree) the bigger the loss. **Thus links between the least connected nodes are the most important (note that they are also the strongest).** For intermediate values of the trade-off we can get situations, where **links between the most connected species –now the weakest links– are the most important.** This requires γ to me large enough –as well as a broad enough distribution of degrees– for second order terms in γ to manifest.

2 Dynamical justification

Here we justify the choice of focusing on the interaction motif shown in the figure. Suppose node activities x_i (e.g. species abundances) follow a Lotka-Volterra dynamical system

$$\frac{1}{x_i}\frac{dx_i}{dt} = r_i - x_i + \sum_j A_{ij}x_j$$

Then equilibrium activities in the coexisting state will read, in vector notations, if $A = (A_{ij})$, $x = (x_i)$ and $r = (r_i)$

$$x^* = (\mathbb{I} - A)^{-1} r = Vr$$

The matrix $V = (\mathbb{I} - A)^{-1}$ not only determines equilibrium, but also the sensitivity of that equilibrium to changes in growth rate vector r which represents abiotic conditions. In particular, to measure the feasibility domain, one starts from a centroid vector r^* and computes the amount of change that it can withstand before at least one node goes extinct. To compute the importance of a given link in the network, we can look at the effect that its removal has on the matrix V. The removal, or more generally the change in interaction between node k and l is a perturbation of the matrix A, that we can denote P. Thus

$$V_P = (\mathbb{I} - A - P)^{-1} = ((\mathbb{I} - A)(\mathbb{I} - VP))^{-1} = (\mathbb{I} - VP)^{-1}V$$

at first order we may approximate the new V-matrix as

$$V_P \approx V + VPV$$

where $P_{kl} = -A_{kl}$, $P_{lk} = -A_{lk}$ and $P_{ij} = 0$ otherwise (if it was just a proportional reduction of the strength of the link we could but $P_{kl} = -\epsilon A_{kl}$ and so forth). Thus the difference matrix δV has elements

$$\delta V_{ij} = -V_{ik}A_{kl}V_{lj} - V_{il}A_{lk}V_{kj} = -\delta V_{ij}^{\leftarrow} - \delta V_{ij}^{\rightarrow}$$

where $\delta V_{ij}^{\rightarrow}$ comes from the perturbation of link from l to k (from right to left in the figure), whereas $\delta V_{ij}^{\rightarrow}$ gives the effect of the opposite direction. Focusing on the former, we have that

$$\delta V_{ij}^{\leftarrow} = V_{ik}A_{kl}V_{lj} \approx \begin{cases} A_{kl} & i = k, j = l \\ A_{ik}A_{kl} & i \neq k, j = l \\ A_{kl}A_{lj} & i = k, j \neq l \\ A_{ik}A_{kl}A_{lj} & i \neq k, j \neq l \end{cases}$$

where we have used a first order expansion of V as $V \approx \mathbb{I}+A$, valid if interactions are not too strong (for mutualistic systems this just means that the equilibrium is not too close to instability). We thus recognize the various contributions identified and computed in the previous section. To compute the importance I

of the link we should compute the magnitude of δV, for instance as the Frobenius norm

$$I = ||\delta V||_F = (\sum_{i,j} \delta V_{ij}^2)^{\frac{1}{2}}$$

since all elements of δV_{ij} have the same sign, however, this norm will be similar to a simple sum of elements

$$I \approx |\sum_{i,j} \delta V_{ij}| = \Delta$$

we are thus left with the quantity Δ computed in the previous section. Note that we could be a bit more precise in our bookkeeping of terms of increasing order in γ by using a second order expansion of V, $V = \mathbb{I} + A + A^2$ which would give

$$V_{kk} \approx 1 + \gamma^2 C_\delta d_k^{1-\delta} + O(\gamma^4);\ V_{ik} \approx A_{ik}\left(1 + \gamma^2 C_\delta (d_i^{1-\delta} + d_k^{1-\delta})\right) + O(\gamma^4)$$

and would permit a rigorous treatment of terms $O(\gamma^3)$ in δV_{ij}. We can guess already that these terms will increase the importance of high degree nodes, due to the powers $d_k^{1-\delta}$ that occurs in the above expression.

3 Generalism, vulnerability and importance

The manuscript by Simmons et al is interested in the role of generalism of a link defined as the average degree of the nodes that it connects

$$D_{kl} = \frac{d_k + d_l}{2}$$

I would be inclined to call this a measure of opportunism of the link, rather than generalism. Indeed, D_{kl} controls the probability, given the number of links that node k and l have, that those two nodes are connected. If they both have many links, it is highly probable that they are connected. Thus this particular connection is not, a priori, special. On the other hand, if both nodes have only few links, the fact that they are connected could suggest something special about their connection. Beyond the semantic issue, what seems intuitive to me is that it is more likely that an opportunistic link could be lost before the nodes would go extinct (a slight change in conditions could make a generalist species change its foraging strategies, whereas this would be harder for a specialist that only has a few partners to choose from).

From the above analysis we have seen that nodes with larger D_{kl} can be the most important, even if they are the weakest. **Here I would say that the most vulnerable links are also the most important. This requires the trade-off δ to be relatively weak but also that mutualistic strength is high enough for the whole motif around the perturbed link to be affected. If the trade off is too strong, or if mutualism is too weak then specialized links are indeed the most important, but that is simply because they are the strongest by construction.**
