# Supplementary material for: Estimating the risk of species interaction loss in mutualistic communities
Source: PLoS Biol. 2020 Aug 31;18(8):e3000843. doi: 10.1371/journal.pbio.3000843 (PMC7485972; doi:10.1371/journal.pbio.3000843)
Supplement: Attachment — Submitted filename: ResponseToReviewers_r2.pdf [file pbio.3000843.s015.pdf]

The decision email contained one comment from the Academic editor regarding the use of the term ‘importance’, an issue that was also raised by Reviewer #3:

"[it] indeed seems a questionable term as it usually has other meanings in ecology, so a clearer, more specific term would be more appropriate."

Response: We agree with the Editor that this term could cause confusion as it has other meanings in ecology. We have therefore replaced the term ‘importance’ with ‘feasibility contribution’ throughout the manuscript and supplementary information as suggested by Reviewer #3.

REVIEWERS' COMMENTS:
Reviewer #1:
[identifies himself as Jean-Francois Arnoldi]
[also see attached PDF]

This is my second review of this manuscript by Simmons et al.

In my first review I had emitted the possibility that the relationship found between link vulnerability and importance was inherent to the definitions of these notions, which discouraged me to dig much deeper in the paper. The authors answered that if there is a connection, it is not a trivial one since it can be strongly weakened by the network complexity (as shown in a supplementary figure showing relationship between link generalism and importance).
I accept this, but this prompted me to investigate this relationship more thoroughly, in a way that I could understand (see attached document).

My initial critique was basically that vulnerability is defined in a way that makes stronger links more vulnerable, and that strong links are inevitably the most important. But maybe I was wrong.

I identified that the core ingredient of the paper is the trade-off (delta in the paper) between the generalism of a species (number of interactions) and strength of these interactions. Precisely, that this trade-off can lead to situations in which weak links are the most important links (with relates to my previous critique and the author's reply).
The way one can see that is by imagining removing a link between generalist species. Although this link is weak, because it connects species that have many links, the effects of the removal will ripple trough many species and can thus have a large impact. Note that this reasoning is quite specific to mutualistic networks where one can add up effects ("the friend of my friend is my friend", whereas for negative interactions: "the enemy of my enemy can be my friend" leads to complications when adding up indirect effects). In the document attached I propose that this occurs if overall mutualism (gamma in the paper) is strong enough, while the trade-off delta exists but is not too strong.
My notes also explain why I think that, if the trade-off is strong, it will inevitably be links between specialist species that will be the most important, simply because they are the

strongest by construction. I suspect that the authors results hold in the latter case, where the strongest links are the most important.

Maybe I am wrong in my analysis (it is indeed, approximate and could be improved), but overall I do think that a clear mathematical explanation of the presented results and their domain of validity is missing from Simmons et al's manuscript.
My notes suggest that such an analysis is not out of reach.

On the topic of the relationship between link vulnerability and generalism, I came to the opposite conclusion from what I understood the authors suggest. A link is said to be "general" if it connects species that already have many links. In other words, the fact that these species are connected is not surprising, given that they have many links. On the other hand, that two otherwise poorly connected species do interact is unexpected, and thus suggests that their interaction is somewhat special. Which of the two kind of links is the most vulnerable (all else being equal)? At first sight, one would say the latter less expected link, which is what the authors propose, based on a paper by Aizen et al (science 2012). But come to think of it, I find this to be very strange. Indeed, maybe the specialist species themselves are more vulnerable, but not the link between them if the two species are present. In the extreme case, if an insect only feeds on one plant, as long as the insect and plant are present this insect will pollinate that plant. On the other hand, if an insect has many plants to choose from, and some of these plants are visited by many other insect species, it seems plausible that a link between the insect and a highly visited plant would be easily lost (e.g. a slight change in behaviour). I am not an expert of the topic so I won't affirm that I am right. But clearly a strong argumentation is needed here. From what I could get from Aizen et al, link extinction is not separated from species extinctions (and frequency of visit is dot defined per capita, see my point bellow). Again I could be wrong, but there is clearly something a bit subtle here that requires some explanation.

The irony is that, by disagreeing twice with the authors, I end up being in agreement with their overall claim: that the most vulnerable species can be the most important. But only if mutualism is large enough and the afore mentioned trade-off is not too strong (I think that, in the authors current view, it would be the opposite conditions that would allow the author's claim to hold).

I recommend that the authors delineate more clearly the domain of validity of their claim, and justify more thoroughly the many assumptions that go into their analysis. In particular, I find the statistical analysis leading to a value of delta very arbitrary. Furthermore, it is based on records of the frequency of insects visits (fij in the text). Maybe I missed something, but those frequencies are likely to be strongly dependent on insect and plant abundance and thus should not directly inform per-capita interactions (which is what the paper is about, at least the method to define link importance). If I am wrong and fij really relates to per-capita interactions, why not use it directly to parametrize link strength? delta would then be a phenomenological parameter used to explain the results (in terms of trade offs as explained above and in my notes). At this point I suggest to treat delta as a free parameter and identify the domains in which vulnerable links are the most

important. Overall this paper should aim at proposing clear hypothesis and not make definite ecological claims, as there are too many parts of the analysis that rely on arbitrary choice of parameters (strength of mutualism, strength of completion, trade off etc...).

Although I am critical, I must say that the paper is very well written and (obviously) thought-provoking. The idea of looking at the effects of perturbing interactions (what we called structural stability in paper with Bart Haegeman from 2016) is something that I personally think is very important and generally overlooked, and I am glad that the authors thought to investigate it. I sincerely hope that this review and attached notes will be helpful.

Response: We thank the Reviewer for their detailed consideration of our manuscript and helpful comments.

There are three main issues raised by the reviewer:

1. The delta parameter is a key parameter that the Reviewer has calculated should affect the relationship between vulnerability and importance (‘importance’ is now termed ‘feasibility contribution’ following comments from the Academic Editor and Reviewer #3). It is therefore necessary to identify the range of delta values in which vulnerable links are the most important (i.e. the range of delta values for which our conclusion holds).

2. Following Aizen et al (Science 2012), we treat links between specialist species as more vulnerable than links between generalist species. The Reviewer suggests that this may only be true because specialist species themselves are more vulnerable, rather than the link itself. If the two species involved in the link are present, the Reviewer argues that generalist links may be more vulnerable than specialist links, because a slight change in behaviour for a generalist species could result in a link being lost, while in a specialist link both species are reliant on each other and so will always interact and the link will not disappear. The Reviewer asks that we explain and clarify this issue more clearly in the text.

3. The Reviewer argues that the way the delta parameter is estimated from empirical data is based on the frequency of insect visits which are dependent on species abundance and thus should not inform per-capita interactions which is what the importance (now called ‘feasibility contribution’) metric requires.

We respond to each of these in turn.

1. We agree with the Reviewer that the delta parameter could influence the relationship between vulnerability and importance. We have therefore repeated our analysis at different values of delta to identify the domain of delta values in which vulnerable interactions are the most important (see figure below).

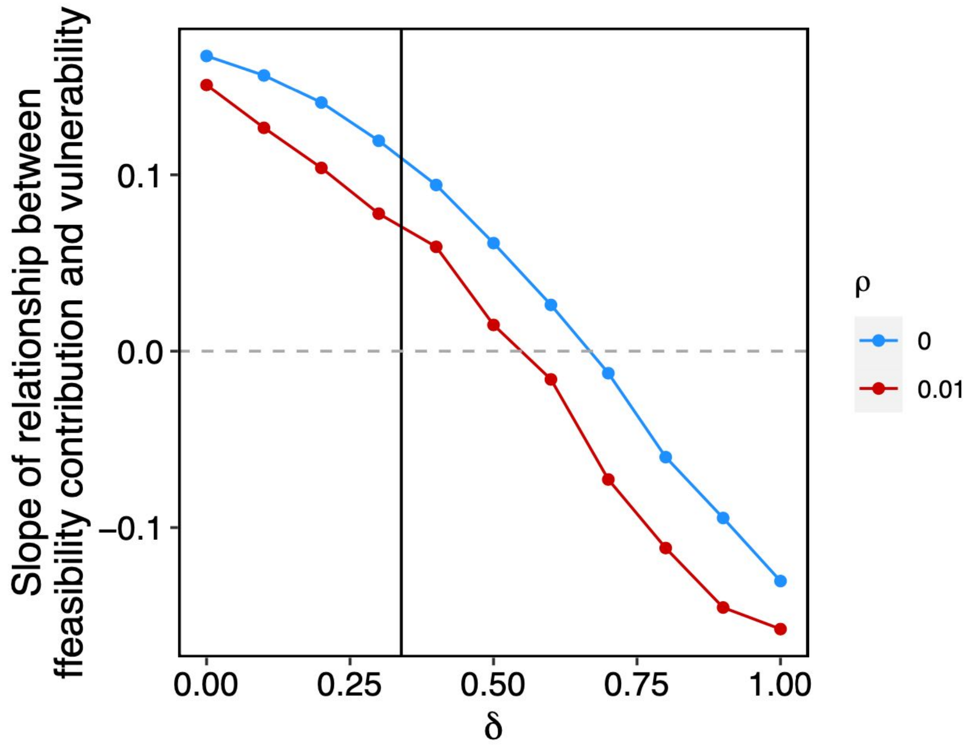

As suggested by the Reviewer, the delta parameter influences the slope of the relationship between vulnerability and importance (feasibility contribution), with qualitatively similar results for both zero (ρ = 0) and weak (ρ = 0.01) competition. Where delta < ~0.6-0.7, vulnerable links are the most important (have the highest feasibility contribution). The vertical black line indicates the value of the empirically-estimated delta value. We believe the empirically-estimated delta value remains valid (see response to point 3 below), and so we present this result in the main text. However, we agree with the Reviewer that it is very important to map the domain of delta where vulnerable links are the most important, and so we have included the above figure in the Supplementary materials, alongside a detailed description of the delta parameter and its meaning (Analysis S4; Figures S8 and S9), so that readers can be fully aware of our analysis' assumptions and how these affect the conclusions, as pointed out by the Reviewer.

2. We agree with the Reviewer that generalist species likely have, on average, less fidelity to their partners than specialist species have to their partners. However, *a priori*, we do not necessarily agree that this means that generalist links are more vulnerable than specialist links. This is because of two other factors that are not discussed by the Reviewer. The first is interaction asymmetry. The Reviewer's example only considers links between two generalist species, or two specialist species. While such interactions do occur, there is also strong evidence that asymmetric specialisation between partners (i.e. a specialist interacting with a generalist) is a prevalent feature of plant-pollinator networks (e.g. https://doi.org/10.1890/03-3112 , https://doi.org/10.1126/science.1123412). In the case of asymmetric links, the presence of a specialist species in the link would decrease the link's generalisation, but could also make the link more vulnerable because, as the Reviewer mentions, the generalist might have little fidelity to the partner and so could switch away at any time. This might then support that specialist links are more vulnerable, as specialisation may come from asymmetry rather than the extreme case of two one-degree species

interacting with each other. (We note that we investigated including asymmetry in our analysis following a comment from Reviewer #4 in the first round of reviews, but we did not include it due to collinearity).

The second factor is species abundance. The Reviewer writes that if two species are present, they interact. However, this discounts the important role of abundance: even if species are present they may not interact, particularly if one or both partners are at low abundance. This is because low abundance makes it harder for species to search and acquire resources. At extreme low levels of abundance, ecological extinction can occur, which is “the reduction of a species to such low abundance that, although it is still present in the community, it no longer interacts significantly with other species” (Estes et al. Conservation Biology. 3: 252-264, 1989). Specialist species tend to have lower abundance than generalist species (https://doi.org/10.1111/oik.06104; https://doi.org/10.1034/j.1600-0587.2003.03443.x ; https://doi.org/10.1890/02-0587 ; https://doi.org/10.1890/07-0451.1). Thus specialist links may be more vulnerable because specialist species, while not extinct, tend to be lower abundance and thus links between them are more likely to be disrupted by climatic, environmental, or biological factors as the species struggle to find each other (e.g. due to changes in weather, obstruction of vision by tall plants etc).

For these two reasons, we do not reach the same conclusion as the Reviewer that generalist links are more vulnerable than specialist links. However, we acknowledge this is a difficult issue to settle a priori, because the Reviewer’s argument about link fidelity being higher among specialist species might suggest that generalist links are more vulnerable, while our arguments about asymmetry and abundance suggest that specialist links are more vulnerable. The Aizen et al (2012) analysis agrees with our conclusion, but as the Reviewer points out, that analysis did not separate link extinction from species extinctions, and thus did not consider link extinction *per se*.

We therefore re-analysed the original Aizen et al (2012) dataset, modifying the analysis to separate link extinction from species extinctions. The Aizen dataset comprises 12 networks from hilltops of varying size (from 12ha to 2147ha). Aizen et al use this size gradient as a proxy for habitat loss, and find that the links present in smaller hilltops are nested subsets of the links present in larger hilltops. They go on to find that the links which survive the habitat loss gradient are those which are generalist.

In our re-analysis, we wanted to make sure that link extinctions were not because the partner species are more vulnerable and went locally extinct, but instead because the link itself was more vulnerable. This meant we had to consider links which met the following criteria:

- The species involved in the focal link must occur in all networks being considered. This removes the possibility of link extinction being a result of species extinction: even if the link is not present, its partner species will be, and so the disappearance of the link will be a consequence of factors other than local-species extinction.

- In networks in large hilltops, the species involved in the focal link must interact. This is because the networks in large hilltops are ones where there has not been any habitat loss. And thus to determine which interactions are lost along the habitat loss gradient, interactions must occur at the start of the gradient. Given that links in smaller networks are significantly nested subsets of the links in larger networks, many links will meet this criterion.

In networks in small hilltops, species involved in the focal link can either interact or not. This then gives us a set of species pairs where i) the species occur in all networks considered, ii) the species interact in the larger network, and iii) the species either interact or don't interact in the smaller networks. This then allows us to examine each species pair, and to see whether or not their interaction ‘survived’ the habitat loss from the larger to the smaller networks. We can then determine the characteristics of the links that ‘survived’ the habitat loss versus those that did not, and test whether the links which survived (i.e. those which were less vulnerable) are those that were more generalist.

Due to our requirement that species pairs must occur in all networks being considered (to remove any effects of species extinction), it was only possibly to conduct our analysis on two networks at a time (one large hilltop, one small hilltop). This is because the more networks that are considered at once, the fewer species pairs occur in all the networks. We therefore considered all possible pairs of large hilltop networks and small hilltop networks. We defined large hilltop networks as those in hilltops greater than 1500ha, while small hilltop networks were defined as those in hilltops less than 600ha. These quantities reflect the distribution of hilltop areas, where a group of networks are clustered in small hilltops, and another group are clustered in large hilltops:

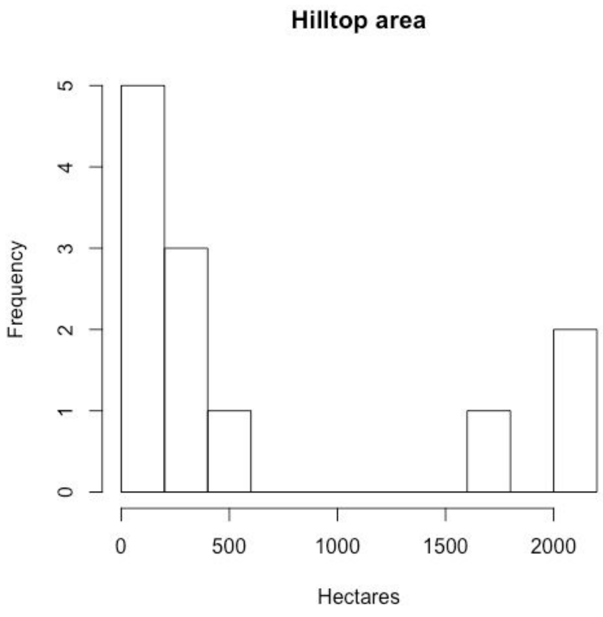

For each pair of small and large hilltop networks, we extracted all species pairs which interacted in the large network, and measured the generalisation of all their links. We then considered the subset of these species pairs which also occurred in the small network. We

used a one-tailed wilcoxon signed rank test to test whether the links which survived the transition from the large to small hilltop networks (i.e. species pairs which interacted in both the large and small network) were those that were more generalised in the large network. Conversely, we expected that links that did not survive the transition (i.e. species pairs which interacted in the large network but not in the small network) were those that were more specialist in the large network. The direction of the tests was informed by the hypothesis that generalisation was higher among the set of links that survived the habitat loss (i.e. that generalisation was higher among less vulnerable links). In total there were 27 different combinations of small and large hilltop networks. The analysis could not be completed for 3 combinations of networks where all species pairs that interacted in the large network, and which also occurred in the small network, also interacted in the small network. In other words, for these three networks, all interactions that met our criteria survived the habitat loss gradient, and thus a comparison of ‘survived’ versus ‘not survived’ links was not possible.

We found that generalisation was significantly higher for links which survived the habitat loss (less vulnerable links) in 79% of network pairs (19/24). Thus links which were generalist in the large network tended to survive the habitat loss, while specialist links tended to not survive. In other words, vulnerable links were those that were significantly more specialised (had significantly lower generalisation). (If we count the 3 networks for which tests could not be completed as being cases where generalisation was not significantly higher for less vulnerable links, then our hypothesis was true in 70% of network pairs (19/27).) We also note that in 96% of network pairs (23/24), mean link generalisation was higher for surviving (less vulnerable) interactions than vulnerable interactions, suggesting that, in these data, there is little support for the Reviewer’s hypothesis that generalised links are more vulnerable.

As we conduct multiple tests, there is an increased probability of incorrectly rejecting the null hypothesis of no change in network metrics (Type I errors). We used the equation given by Moran (2003; https://doi.org/10.1034/j.1600-0706.2003.12010.x), based on a Bernoulli process, to calculate the probability of a given number of significant tests from a given number of trials. The probability, p , is given by the equation
P = [N!/N(-K)!K!] * alpha^K * (1-alpha)^(N-K)
where N is the number of tests conducted, and K is the number of tests below the significance level α.
We found that the probability of obtaining our results by chance alone was 6.27e-21. Therefore, despite the inflated Type I error rate resulting from multiple tests, the number of significant results we found was substantially greater than expected from chance alone

From this we conclude that our original definition of vulnerable links being more specialised remains true, even when controlling for species extinctions. We have included this analysis in the supplementary material along with a brief outline of the arguments (both ours and the Reviewer’s) that we discussed in this response (Analysis S3).

3. The Reviewer is right that delta was estimated using empirical information on the frequency of insect visits. Our method for empirically estimating the value of delta was taken from the paper by Rohr (2014, Science), where the feasibility measure was introduced for

mutualistic networks. In this paper, they address the issue of using frequency of visits as a proxy for per-capita effects. They say “Thus, qij is the observed number of visits of animal species j on plant species i. This quantity has been proven to be the best surrogate of per capita effects of one species on another (D. P. Vázquez et al., Species abundance and asymmetric interaction strength in ecological networks. Oikos 116, 1120–1127 (2007). doi: 10.1111/j.0030-1299.2007.15828.x)”. (https://doi.org/10.1111/j.1461-0248.2005.00810.x). Thus we are confident in the method used to estimate delta empirically. We have added the above references to the main text as justification for using the observed number of visits as a surrogate of per capita effects (L425-426 in the clean version of the revised manuscript). However, by including the analysis from our response to point 1 above in the supplementary information (Analysis S4, Figures S8, S9) , if reader’s disagree with the empirical delta value, they can now clearly see the domain of delta values where more vulnerable links are more important. In this way, as the Reviewer suggests, we are open about our assumptions and provide this information to readers.

Reviewer #2:
[identifies himself as Shawn J. Leroux]

The authors have done an outstanding job of addressing the many reviewer comments. The additional details in the methods (including some new analyses) and revised introduction and discussion have greatly improved the ms. I have no additional concerns.

Response: We are glad the Reviewer is satisfied with the revisions and we thank him for his kind comments about our manuscript.

Reviewer #3:

I appreciate the effort that the authors have put into answering the concerns I raised in my previous review. The manuscript is very well written and there is merit in what the authors present in this manuscript. Moreover, I was pleased to see the changes made to the manuscript, including new figures, clarifications and additional discussion points provided by the authors.

While some of my concerns regarding how much the authors' "importance" actually reflects ecological importance remain, I understand that using simulations to understand the identity of affected species due to the vulnerable link removals might not be a trivial exercise. My concerns are perhaps little more than a matter of terminology. Namely, I am still troubled by the use of the word "Importance" to define the 'contribution to' a well known network property. I think it's a bit misleading, and I *personally* feel that something along the lines "contribution to feasibility" would be much more appropriate. That said, I appreciated the clarifications added by the authors across the manuscript regarding this point.

Response: We thank the Reviewer for their positive comments about the manuscript. We agree with the Reviewer that the term ‘importance’ could cause confusion. We have therefore replaced the term ‘importance’ with ‘feasibility contribution’ throughout the manuscript and supplementary information as suggested by the Reviewer.

Reviewer #4:
[identifies himself as Ignasi Bartomeus]

Thanks for the super detailed response and the extra analysis, which makes the manuscript very robust. I don't have any further comments and I congratulate the authors on this nice study.

Response: We are glad the Reviewer is satisfied with the revisions and we thank him for his kind comments about our manuscript.
