# Supplementary material for: Estimating the risk of species interaction loss in mutualistic communities
Source: PLoS Biol. 2020 Aug 31;18(8):e3000843. doi: 10.1371/journal.pbio.3000843 (PMC7485972; doi:10.1371/journal.pbio.3000843)
Supplement: Attachment — Submitted filename: PLOS Biol revision 3 reviewer response.pdf [file pbio.3000843.s016.pdf]

Editor comments:

Based on the remaining review, we will probably accept this manuscript for publication, assuming that you will modify the manuscript to address the remaining points raised by the reviewer. Note that while the reviewer marks some of his points as "mere suggestions," we urge you to address them as fully as you can. Please also make sure to address the data and other policy-related requests noted at the end of this email.

1. Response: We confirm that we have addressed all the Reviewer comments, including those marked as suggestions. We have also addressed all the data and other policy-related requests.

Reviewer comments:

The authors did a great job at addressing my previous comments. In particular, the analysis of the cited paper by Aizen et al (science 2012) is quite helpful.

2. Response: We thank the Reviewer for their positive comments and are glad they found the additional analyses helpful.

Furthermore, the new figure where delta is varied is indeed the kind of results that I though was missing in the previous version. Note that it could be useful to see the impact of gamma (strength of mutualism, here it is defined so that the dominant eigenvalue of the interaction matrix is -0.5, but one could look at cases where this eigenvalue is -0.9, weak mutualism, and -0.1, strong mutualism, for instance).

3. Response: We thank the Reviewer for this suggestion, and we have now repeated our analysis for average mutualistic strength set to 10%, 50% and 90% of the average mutualistic strength at the stability threshold (Figure B in S4 Analysis and see figure in response #4 below).

My remaining comments are the following, since other reviewers were already satisfied with the previous version I provide those comments as mere suggestions:

In the new figure where delta is varied, I don't think that it makes sense to compute vulnerability, since it requires the frequency of visits (fij fixed from observations) which in turn ought to determine delta (which is now varied). Thus I would suggest to plot the slope of specialism in contribution to feasibility against delta.

4. Response: We have updated the figure where delta is varied (Figure B in S4 Analysis and below). As suggested by the Reviewer, the response variable is now the slope of the relationship between link specialism (termed link generalisation in our analysis) and contribution to feasibility.

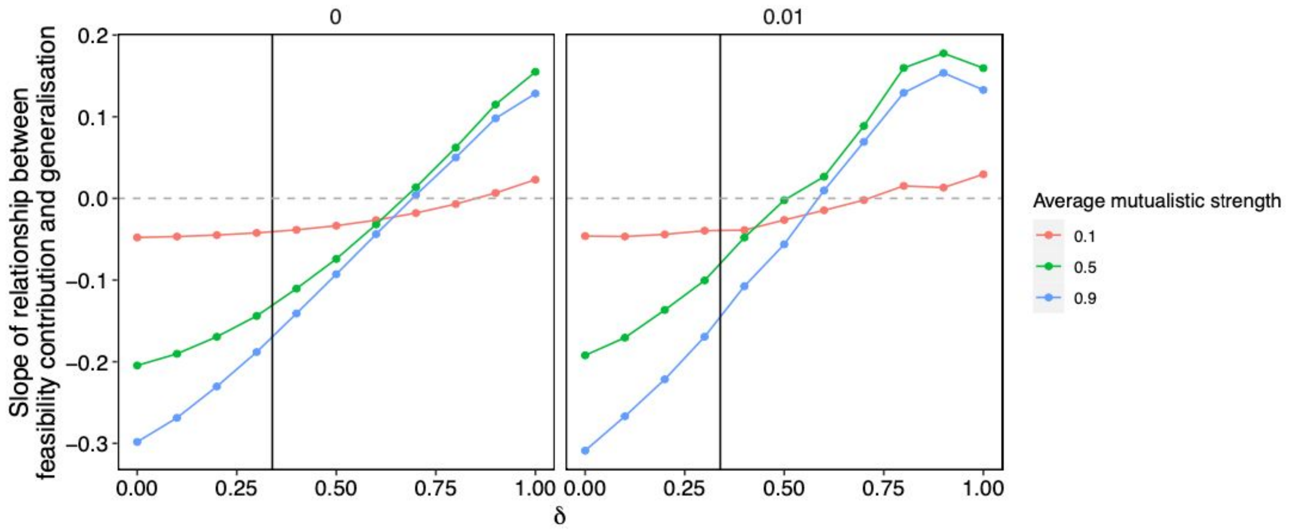

Each line is a different average mutualistic strength value. 0.1 means average mutualistic strength was set at 10% of average mutualistic strength at the stability threshold, and so on. The two panels are for two values of intraspecific competition (left rho = 0, right rho = 0.01). The vertical line is the empirical delta value.

This new figure shows that only for high delta values (~>0.7) the slope is positive, whereas for most values of delta the slope is negative. Importantly, at the empirical delta value, the slope is negative at all mutualistic strengths, so our results are not sensitive to mutualistic strength in terms of directionality of the slope.

I am also surprised that the slope is positive and strongest when delta is zero (no generalist-strength tradeoff), as I would naively expect the opposite. I encourage the authors to double check their simulations and see if they can provide an explanation.

5. Response: The Reviewer was surprised that, in the previous version of the delta sensitivity analysis, the slope between vulnerability and feasibility contribution was positive and strongest when delta was zero. The Reviewer expected the opposite - that at delta zero the slope would be at its most negative.

We believe this discrepancy can be explained by the fact that the vulnerability metric which we used in the previous version of the plot has an opposite direction to generalisation which we use in the current version above: more generalised links are less vulnerable and less generalised (more specialist) links are more vulnerable. Thus the positive slope between vulnerability and feasibility contribution when delta was zero is equivalent to a negative slope between generalisation and feasibility contribution when delta was zero.

In the new delta sensitivity plots above (response #4), that were requested by the Reviewer, at delta zero the slope between generalisation and feasibility contribution is at its most negative, as expected by the Reviewer. We have clarified this issue in the text accompanying this analysis (S4 Analysis, below Figure B).

It would be worthwhile to see if the results are specific to the empirical networks or would also hold for a random bipartite network with comparable variance in number of links per nodes.

6. Response: We followed the reviewer's suggestion and repeated the analysis for an ensemble of random bipartite networks with comparable degree distributions. We chose to generate random networks that had a size and connectance the same as the median size and connectance of networks in our dataset (47 species, 0.16 connectance). We used the curveball algorithm (https://doi.org/10.1038/ncomms5114) to produce 50 randomisations of the network from our dataset which had this median size and connectance. The curveball algorithm randomised the structure of this empirical median network while maintaining its degree distribution to ensure the random networks were comparable (https://doi.org/10.1038/ncomms5114). The results are shown below:

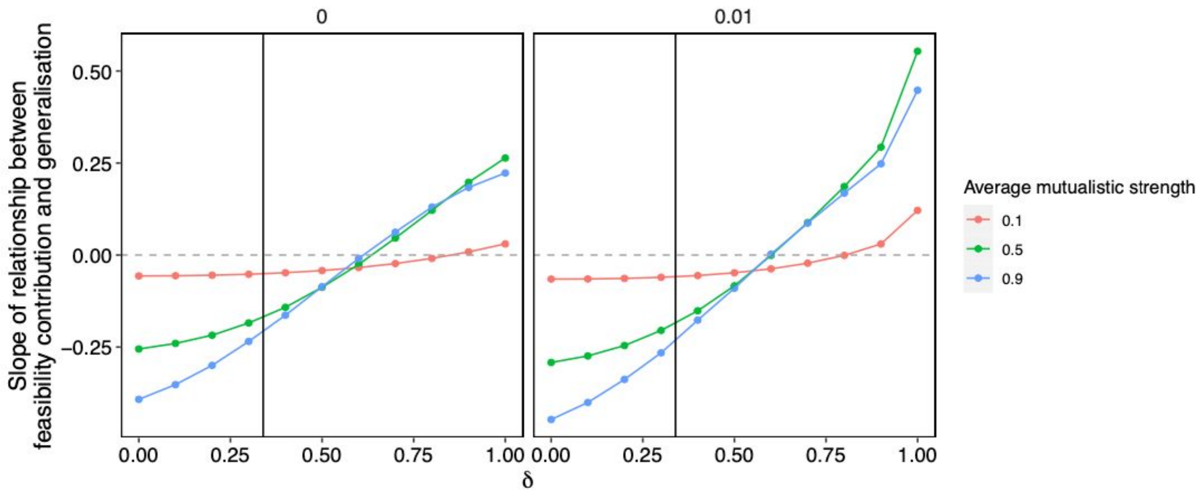

As can be seen, the results from the random networks are qualitatively the same as the results from the empirical networks. This implies that the degree distribution determines the broad pattern of how the slope between generalisation and feasibility contribution varies with delta and mutualistic strength, and confirms that our result is robust for other bipartite networks that resemble the structure of empirical mutualistic communities. This analysis is now included in the supplementary information (Figure C in S4 Analysis).

Finally, I am still not convinced by the use of frequency of visits in the analysis. Indeed, maybe it can be used to infer the tradeoff delta (although I don't really get it), but it is clear that this frequency depends on abundances. This is not consistent with the feasibility analysis that starts from a growth rate vector where all species have the same abundances and then looks for the size of perturbations of that growth rate vector that leads to extinctions (and thus varies abundances widely, so frequency of visits can not be fixed). I thus encourage the authors to provide results on specialism vs contribution to feasibility instead of the empirically constructed measure of vulnerability of Aizen et al.

7. Response:

The reviewer is right that visitation frequency may depend on species abundances (for which we do not have data for the networks we analyse). On the other hand, feasibility is calculated in such a way that the domain which represents the parameter space within which all species survive, occurs for any combination of abundances. This means that for, let's say, 5 points in the feasibility domain, these points can all have very different combinations of species abundances, which implies that the visitation frequencies (that may depend on abundance) should be different among these 5 points. In our case, however, the empirically-measured visitation frequencies correspond to a single combination of abundances (abundances that we do not know, however).

Although this creates an apparent confusion of whether we can correlate the 2 measures (vulnerability and feasibility), we do not believe this is a problem. The empirical visitation frequencies (which may be related to abundances - but abundances we do not know) correspond to a single point within the feasibility domain, or perhaps to a point outside the feasibility domain. In other words, as we do not know the actual abundances of the species in our empirical networks, we can only infer, based on the fact that these communities occur in nature, that the empirical communities are at a single point within the feasibility domain. So what the feasibility analysis after the removal of a focal link actually gives us, is the probability that the network without this link is still feasible or not. If the feasibility domain gets bigger, there is a higher probability that the empirical network – with its particular combination of frequencies and (unknown) abundances – is within the feasibility domain without the focal link. If the feasibility domain gets smaller, it is less likely that the network is within the feasibility domain without the focal link.

Of course, ideally, we would like to have a measure of visitation frequency that is independent of, or corrected for, species abundances so that we have a more accurate measure that would remove any concern similar to the one raised by the Reviewer. Although visitation frequency may be influenced by species abundances, it may not represent the true abundance because it is not independent of the network itself (Vasquez et al 2009, Ann Bot). The few studies looking at the relationship between the distribution of species abundances and visitation frequency provide mixed results. Olito and Fox (2014, Oikos), find that abundances do not successfully predict observed interaction frequencies. Hu, Dong and Sun (2019, PLOS ONE) find that abundance explains only 20-40% of variation in pairwise interaction frequencies, and Vizentin-Bugoni et al (2014, Proceedings B), find that "frequency of interaction is a poor proxy for abundance".

We felt that this mixed picture of the extent to which visitation frequency is affected by abundance warrants further investigation so we attempted to investigate it using an updated version of the hummingbird-plant pollination network from the Vizentin-Bugoni et al (2014) paper, which comes with independent abundance data.

We correlated the observed visitation frequency with the visitation frequency that would be expected from abundance alone. To generate the abundance-driven frequencies we followed the standard technique of Vázquez et al (2009, Ecology), and created a probability matrix where the probability of each species interacting equalled the product of their relative

abundances. Thus abundant species pairs are more likely to interact than rare species pairs. We then used a negative binomial model to correlate observed visitation frequency of each species pair against the corresponding probability of interaction that would be expected from abundance alone. We found a significant (P = 0.016), but very weak (McFadden Pseudo-R^2 = 0.01), correlation between abundance-driven probability of interaction and observed interaction frequency (S5 Analysis and figure below - 'Empirical' panel).

We then went a step further to test whether this weak relationship was a peculiarity of our data by repeating the analysis, using idealised abundance distributions (exponential, lognormal, Fisher log series, uniform) instead of the empirical abundance distribution. We found similar results for the idealised abundances as we did for the empirical abundances. Fisher log series and exponential abundance-driven interaction probabilities were significantly related to empirical interaction frequency (P = 0.03 and 0.03 respectively), but for lognormal and uniform no significant relationship was found. For the two significant relationships, McFadden pseudo-R^2 remained very low: 0.006 for exponential and 0.006 for Fisher log series.

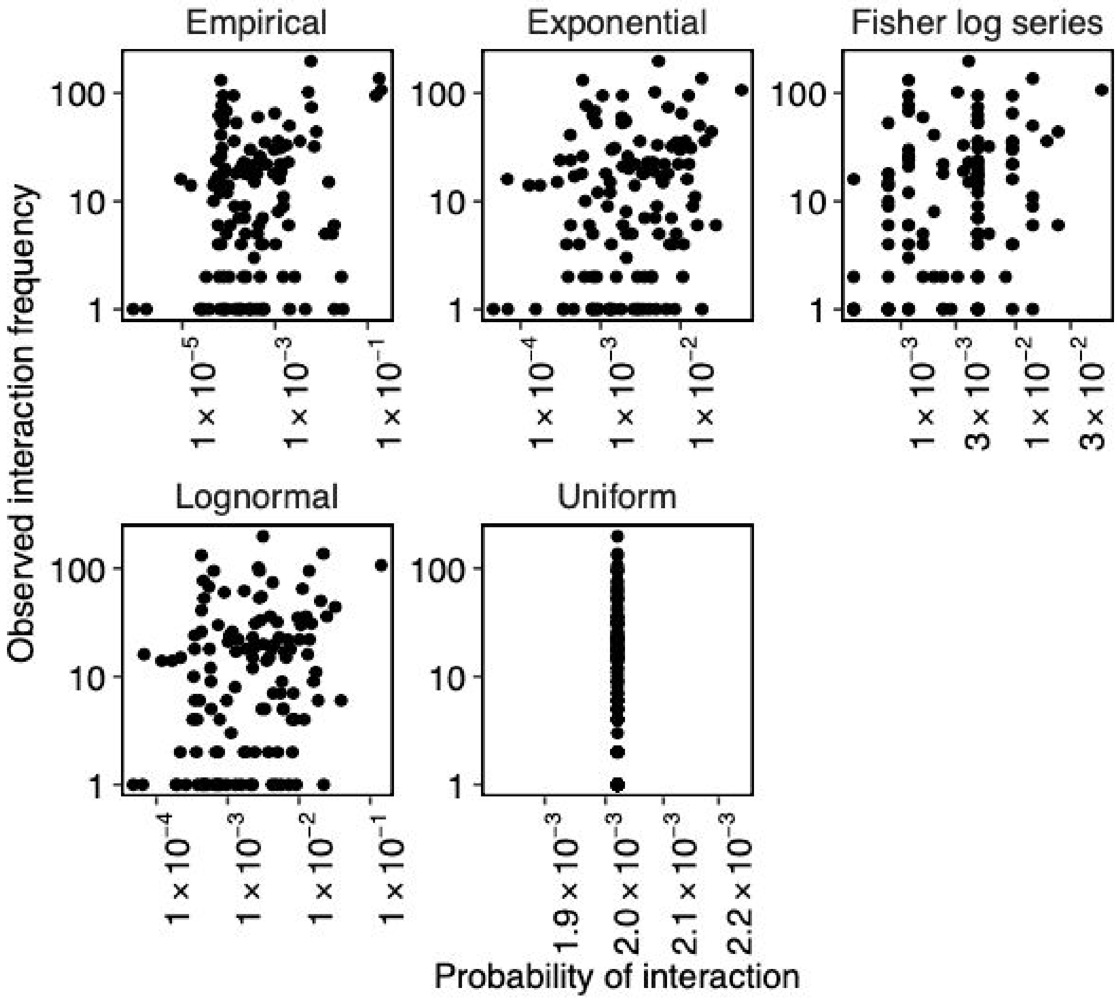

Fig A: Species were assigned an abundance from each distribution based on their percentile i.e. a species in the 88th percentile in the empirical abundance data was assigned the corresponding 88th

percentile value from the idealised distribution. We considered four possible distributions: exponential, fisher log series, uniform and lognormal. The exponential distribution had a rate of 1. The Fisher log series requires parameters for the number of species S, the total number of individuals N, a measure of biodiversity alpha, and the Fisher parameter y which controls the shape of the distribution. Following de Aguiar (2019, PeerJ), we set S equal to the number of species in the network, and y = 0.5 (the value of y does not matter as we later convert to relative abundances). Using these values, we solved for alpha and N. The lognormal had a mean equal to the mean of the empirical abundances, and a standard deviation of 1. All null abundances were converted to relative abundances.

Interestingly these results suggest that abundance may be a poorer predictor of visitation frequency than traditionally thought. To some extent they also suggest that our correlation between vulnerability and feasibility might not be biased by the visitation frequency uncorrected for abundances.

We have added this new analysis to the supplementary material (S5 Analysis). We have also added a discussion of these issues to the main text (L460-500 main text with all changes accepted). Finally, our entire analysis has been repeated with generalisation (rather than frequency) as the explanatory variable (S2a Figure and S4d,e,f Figure) as requested by the Reviewer.
